# Beatboxers and Guitarists Engage Sensorimotor Regions Selectively When Listening to the Instruments They can Play

**DOI:** 10.1093/cercor/bhy208

**Published:** 2018-08-31

**Authors:** Saloni Krishnan, César F Lima, Samuel Evans, Sinead Chen, Stella Guldner, Harry Yeff, Tom Manly, Sophie K Scott

**Affiliations:** 1 Institute of Cognitive Neuroscience, University College London, 17 Queen Square, London, UK; 2 Department of Experimental Psychology, University of Oxford, Anna Watts Building, Radcliffe Observatory Quarter, Oxford, UK; 3 Instituto Universitário de Lisboa (ISCTE-IUL), Avenida das Forças Armadas, Lisboa, Portugal; 4 Department of Psychology, University of Westminster, 115 New Cavendish Street, London, UK; 5 Graduate School of Economic and Social Sciences (GESS), University of Mannheim, Mannheim, Germany; 6 Get Involved Ltd, 3 Loughborough Street, London, UK; 7 MRC Cognition and Brain Sciences Unit, 15 Chaucer Road, Cambridge, UK

**Keywords:** auditory perception, dorsal stream, expertise, fMRI, musician

## Abstract

Studies of classical musicians have demonstrated that expertise modulates neural responses during auditory perception. However, it remains unclear whether such expertise-dependent plasticity is modulated by the instrument that a musician plays. To examine whether the recruitment of sensorimotor regions during music perception is modulated by instrument-specific experience, we studied nonclassical musicians—beatboxers, who predominantly use their vocal apparatus to produce sound, and guitarists, who use their hands. We contrast fMRI activity in 20 beatboxers, 20 guitarists, and 20 nonmusicians as they listen to novel beatboxing and guitar pieces. All musicians show enhanced activity in sensorimotor regions (IFG, IPC, and SMA), but only when listening to the musical instrument they can play. Using independent component analysis, we find expertise-selective enhancement in sensorimotor networks, which are distinct from changes in attentional networks. These findings suggest that long-term sensorimotor experience facilitates access to the posterodorsal “how” pathway during auditory processing.

Our engagement with music is shaped by a variety of factors, such as our mood, the setting, the piece itself (Blood and Zatorre 2001; Zatorre and Salimpoor 2013), or one’s familiarity with the piece and the genre (Leaver et al. 2009; Dick et al. 2011). It could also depend on whether we can play the instrument the music is produced with. Playing a musical instrument requires one to learn execute complex and highly specific motor movements, often involving multiple effectors, and to use both auditory and somatosensory information to guide these actions. Although we think of learning to play an instrument as a motoric skill, such musical training also results in specific changes in auditory perception and attention (Carey et al. 2015). Short-term training studies also demonstrate that the neural regions recruited during auditory perception change when people have motor experience rehearsing the music they listen to (D’Ausilio et al. 2006; Lahav et al. 2007). More generally, cross-sectional studies indicate that classical musicians recruit regions in the dorsal stream to a greater extent than nonmusicians during auditory perception (Zatorre et al. 2007). However, it is unclear whether experience playing different instruments with different motor effectors leads to different functional specializations within musicians, or whether such change is linked to ear training, (for example, being able to predict typical chord progressions. To address the issue of effector and instrument-specificity within musicians during perception, we examine neural responses during musical listening in 2 groups of nonclassical professional musicians, beatboxers and guitarists, as well as nonmusicians.

Beatboxers are a group of musicians who use their vocal tract to produce polyphonic music, during which they often closely mimic musical instruments. Humans are one of the only primate species to show highly flexible mastery of the vocal tract, but previous exploration of complex vocal behavior has relied on tasks that involve speech or language, or skills that typically incorporate linguistic elements (e.g., songs that involve words). Beatboxing is independent of these linguistic elements. Also, unlike most other kinds of musicianship, beatboxing is typically not a formally taught skill. It therefore offers an intriguing model of vocal expertise that has never been systematically studied. Guitarists, in contrast, offer different insights into auditory-motor learning, as they produce sound via rapid co-ordinated movements of both hands on a stringed instrument. Studying these musicians does not entail the effects of a shared body of formal musical knowledge, unlike the study of classical musicians. The beatboxers and guitarists also represent cultural and social groups that differ somewhat from typically studied classical musicians. Classical musicians typically start training very early in life (Corrigall and Schellenberg 2015), are generally from higher socioeconomic backgrounds (Müllensiefen et al. 2014), and their training has been found to be associated with increases in attention and IQ (Schellenberg 2006). Nonclassical musicians have been successfully studied before to elucidate the neural correlates of improvisation. Research examining jazz musicians has shown distinct signatures linked to their musical expertise, such as recruitment of the left supramarginal gyrus in rhythm perception tasks (Herdener et al. 2014), changes in mismatch negativity amplitudes linked to overall sound sensitivity (Vuust et al. 2012), and increased sensorimotor connectivity in improvisation tasks (Limb and Braun 2008; Pinho et al. 2014). Here, by studying beatboxers and guitarists in addition to nonmusicians, we examine the extent to which neural changes linked to musicianship are selective to the domain of sensorimotor expertise (vocal tract vs. hands). This design also allows us to probe 2 other issues: 1) whether neural signatures of musical expertise are observed in nonclassical musicians and 2) the extent to which expertise-related changes generalize to unlearned musical skills and untrained musical pieces.

Current models of auditory perception suggest that anteroventral stream regions are important for object recognition and identification (the ventral stream extends from primary auditory regions to the anterior temporal poles), whereas regions in the posterodorsal stream (the dorsal stream extends from primary auditory cortex to the inferior parietal cortex and premotor cortices) are relevant for spatial processing and calculating auditory-motor transformations (Rauschecker and Scott 2009; Lima et al. 2016). A hallmark of classical musical expertise is greater activity in dorsal stream regions when listening to music (Zatorre et al. 2007), and this activity is thought to reflect long-term learning of associations between a sequence of motor actions and a sound stream. This modulation of dorsal stream regions has been demonstrated in short-term training studies of novices learning piano music (Lahav et al. 2007; Chen et al. 2012; Herholz et al. 2015), studies examining musicians versus nonmusicians (Chen et al. 2008b; Grahn and Rowe 2009), and in studies that have examined motor cortex excitability in musicians (D’Ausilio et al. 2006; Rosenkranz et al. 2007). However, these studies have either grouped together different kinds of classical instrumentalists (Chen et al. 2008a; Grahn and Rowe 2009), or studied responses in only one musician group (Bangert et al. 2006; D’Ausilio et al. 2006; Gebel et al. 2013; Kajihara et al. 2013), comparing activity in musicians to nonmusicians. This does not allow us to address whether dorsal stream activity is related to instrument-specific sensorimotor repertoires in musicians (e.g., such differences are seen in the domain of dance, Calvo-Merino et al. 2005). If differences in dorsal stream activity are driven by sensorimotor knowledge and experience, we would predict that musicians who use different motor effectors to play their instruments might show distinct profiles of dorsal stream engagement, as they have different sets of motor expertise and auditory-motor repertoires. Our comparison between beatboxers, guitarists, and nonmusicians during musical listening represents a strong test of the hypothesis that activity in these regions is tightly linked to previous instrument-specific sensorimotor experience, as we contrast brain activity in musicians who produce music with different effectors (vocal tracts vs. hands respectively). Studies that have examined the structural neural basis of instrument-specific expertise provide suggestive evidence for this prediction. For example, instrument-specific differences are noted in the motor cortices in keyboard and string players (Bangert and Schlaug 2006), as well as the tracts connecting auditory and motor cortices (Halwani et al. 2011; Ruber et al. 2015). Studies of musical production also suggest instrument-specific effects on brain regions, such as increased responses in larynx motor cortex for opera singers, and finger representations for instrumentalists (Elbert et al. 1995; Kleber et al. 2010). Finally, an fMRI study of classical musicians, comparing 7 flutists and 9 violinists listening to a trained piece, also provides some support for our prediction (Margulis et al. 2009).

Although our hypothesis is that dorsal stream regions are key regions supporting instrument-specific responses during music perception, previous research does suggest that instrument-specific responses are not confined to sensorimotor regions (Pantev and Herholz 2011). In studies of classical musicians, such responses have been observed in primary auditory regions, and extending down the anteroventral stream. When magnetoencephalography was used to measure the brain responses of violinists and trumpeters, timbres close to those of the musicians’ principal instrument were associated with enhanced auditory representations (Pantev et al. 2001). In a small-sample electroencephalography study, Shahin et al. (2008) demonstrated that violinists and pianists showed enhanced gamma band activity for timbres closest to the instruments they played. This would be consistent with sources in auditory and primary auditory cortices, suggesting that these regions become tuned to specific features of the trained instrument. In addition, in the right posterior superior temporal sulcus, left planum temporale and left anterior superior temporal gyrus, violinists showed greater activity for violin music than actors did (Dick et al. 2011). Margulis et al. (2009) also report an instrument-specific response in the left posterior superior temporal sulcus in a small group of violinists and flutists listening to the same musical piece performed by their own instrument. However, instrument-specific responses in these early auditory regions are unlikely to reflect sensorimotor expertise. Instead, such activity possibly reflects the enhanced experience experts possess identifying various spectrotemporal and musical properties of these different sounds (Angulo-Perkins et al. 2014). The posterior superior temporal gyri bilaterally might be spontaneously engaged in finer auditory categorization for behaviorally relevant sounds. Regions that are placed more anteriorly in the anteroventral stream, such as the anterior superior temporal gyrus, are thought to play a role in speech intelligibility (Scott et al. 2000), and may be involved with the recognition and prediction of sequential auditory input.

An alternative and nonmotoric account of enhanced activity in dorsal stream regions (such as inferior frontal and parietal cortex) during perception is that activity in these regions reflects domain-general attention or executive control processes, in a manner that is not necessarily instrument-specific. Activity in brain regions such as primary motor cortex, inferior frontal gyrus and the inferior parietal cortex is influenced by attention during listening (Dhanjal et al. 2008a; Wild, Davis, Yusuf, et al. 2012; Möttönen et al. 2014). Classical musicianship is often associated with domain-general increases in attention (Strait et al. 2010; Carey et al. 2015) and top-down executive control (Koelsch et al. 1999). It is thus possible that long-term improvements in domain-general perception and attention are responsible for the recruitment of sensorimotor regions during listening in classical musicians (but see Baumann et al. (2008)). If this is the case, we would expect to see musicians recruit dorsal stream regions, but not in an instrument-specific manner. Indeed, instrument-specific expertise effects in dorsal stream regions and a lack of instrument-specific effects in auditory areas would suggest that sensorimotor experience with an instrument, and not domain-general attention, enables access to motor representations during perception. Highly effector-specific responses during perception, such as beatboxers showing increased activity in mouth regions, and guitarists in hand regions, would also indicate more specific sensorimotor access, rather than broad and unspecific attentional activity. However, one possibility is that we would also observe instrument-specific modulation of attentional and executive control networks. This might indicate that the origin of the activity was derived from attentional expectancies created from long-term sensorimotor experience (see Lima et al. 2016, for further discussion on why sensorimotor activity might be recruited during auditory perception). Attention could be specifically tuned towards sounds that musicians have practiced and are familiar with, as they will be able to make stronger predictions about these sequences. Indeed, some researchers have suggested that it is specifically the sensorimotor aspects of musical training that could strengthen overlapping neural networks for attention and cognition. For instance, a recent study suggested that rhythmic expertise, built via long-term percussion training, shaped attentional and inhibitory control (Slater et al. 2018). Others have suggested that long-term instrument training could lead to an automation of task-specific cognitive processes, for instance, those involved in creating new musical sequences and combinations (Pinho et al. 2014). These authors demonstrated that improvisation training in piano players was associated with lowered demands on executive networks, and greater connectivity in sensorimotor networks (also see Limb and Braun 2008).

Here, we use a three-pronged approach to try and disentangle spontaneous sensorimotor activity from responses that could relate to domain-general attentional processes. First, our task involved naturalistic music listening, with no overt task to perform. This minimizes the engagement of domain-general regions that are associated with attention, executive control, and monitoring (Hall et al. 2000; Vannest et al. 2009). Second, we use novel pieces of music which are selected to highlight expertise in different forms of beatboxing and guitar playing, that are unfamiliar to all participants. The use of novel pieces should avoid the dorsal stream activation related to rehearsal/familiarity reported in short-term training studies (Lahav et al. 2007). Third, we use a multivariate approach to characterize the brain networks involved in music perception. Task-based independent component analysis (ICA) approaches have been extensively used to characterize networks that are associated with executive control and attention (Leech et al. 2011), in both visual paradigms as well as those involving listening to auditory stimuli (Braga et al. 2013) or producing speech (Geranmayeh et al. 2014; Simmonds et al. 2014). It is therefore the perfect method for exploring the interplay between expertise and attention as evidenced at the neural level, but has thus far not been used to study effects of instrument-specific expertise. ICA takes advantage of inherent fluctuations in fMRI activity to identify independent spatial networks, which are robust across task and rest. In task-based ICA, we relate the timecourse of spatiotemporal networks to the task design, and assess how each network is influenced by task and subject-factors. We can consequently identify networks typically associated with sensorimotor skill, attention and executive control, and identify which of these networks are influenced by experience. By using an ICA-based approach, we can ascertain if 1) musicians show differential activity in domain-general attentional and executive control networks relative to nonmusicians and 2) whether this is true for both musical styles or specific to the one they are expert in. In addition, we have also used a battery of behavioral measures (Table 1) to assess and control for any domain-general effects of expertise.

**Table 1.** Participant characteristics

	Nonmusicians	Guitarists	Beatboxers
Age	27.8 (8.9)	30.0 (7.8)	26.8 (5.8)
**Age of onset**	n/a	11.2 (2.3)	14.0 (3.8)
**Musical training (years)**	0.03 (0.1)	5.9 (3.9)	3.1 (4.1)
Professional experience (years)	n/a	8.7 (7.3)	8.2 (5.3)
Amateur experience (years)	n/a	11.6 (7.2)	11.3 (8.0)
Pure tone average	1.3 (4.0)	1.7 (3.6)	2.7 (5.1)
*Cognitive tests*			
Matrix reasoning ability (scaled)	60.4 (4.7)	57.9 (6.9)	58.2 (7.3)
Working memory	12.8 (3.2)	12 (3.2)	11.7 (3.5)
*Perception tests*			
** Metric judgment**	25.2 (5.5)	29.9 (0.4)	27.2 (3.8)
** Rhythm discrimination**	24.1 (3.2)	27.1 (2.5)	26.2 (2.5)
** Frequency discrimination threshold**	10.6 (7.3)	4.8 (4.4)	8.0 (3.9)
Duration discrimination threshold	28.6 (10.8)	25.3 (13.2)	27.0 (9.3)
*Musicality: Goldsmiths Musical Sophistication Index*			
** Active engagement**	32.4 (11.6)	50.3 (4.2)	50.5 (6.4)
** Perceptual abilities**	42.6 (6.6)	56.8 (4.0)	51.8 (7.8)
** Musical training**	13.3 (6.3)	43.1 (5.1)	34.9 (6.3)
** Emotions**	31.8 (4.1)	37.2 (3.5)	35.8 (4.6)
** Singing abilities**	22.0 (8.0)	38.1 (6.1)	35.1 (8.8)
** General sophistication**	55.1 (14.1)	105.9 (9.0)	100.6 (13.6)

Means and standard deviation for each group are indicated. Measures where there are significant differences between groups are in bold. Data from one beatboxer were not collected for the metric and rhythm judgment tasks, leaving an *N* = 19. Data from a different beatboxer for frequency and rhythm discrimination tasks were lost due to a technical issue, again leaving an *N* = 19 for these measures from the beatboxers. Additionally, data from one nonmusician were excluded from the frequency discrimination task as it was >3 standard deviations from the average threshold of this group.

Our hypotheses are 1) different types of musical experience will manifest in distinct profiles of sensorimotor engagement during auditory perception, particularly within the dorsal auditory stream. On the basis of studies that indicate that listening to hand and mouth sounds produces separable somatotopic activation in premotor cortex (Gazzola et al. 2006), we further predict that 2) beatboxers and guitarists will show greater activity in “mouth” and “hand” motor regions, respectively. This would indicate that motor experience plays a specific role in forming perception–production links, as predicted by the associative learning account (Heyes 2010). Finally, when using a multivariate approach to characterize the functional networks involved in music perception, we expect 3) expertise-driven effects in sensorimotor networks rather than attentional ones.

## Materials and Methods

### Participants

We scanned 20 guitarists (2 female), 20 beatboxers (3 female), and 20 nonmusicians (2 female) with no history of neurological or audiological disorders. The UCL Research Ethics Committee approved this study. All participants provided written informed consent prior to participation.

Musicianship was defined by a mandatory minimum of 4 years’ experience, which included 1) performing at a professional level for at least 2 years and 2) at least 2 years more of training or amateur experience beatboxing or playing the guitar. On average, guitarists had 8.7 years of professional experience (range: 2–30 years) and 11.6 years of amateur experience (range: 5–29 years). Beatboxers had 8.2 years of professional experience (range: 2–25 years) and 11.3 years of amateur experience (range: 3.5–30 years). None of the guitarists could beatbox. Although 5 of the beatboxers did have some guitar experience, they primarily identified themselves as beatboxers. As this was a much smaller population than guitarists, these beatboxers were retained in the sample. We also found that guitarists tended to start playing their instrument earlier than beatboxers, and received more formal musical training (Table 1).

To ensure that the groups were comparable in general cognitive and hearing abilities, all participants completed a hearing test and a set of cognitive tests (Table 1). None of the participants had hearing loss (the average of hearing thresholds at 500, 1000, and 2000 Hz was <20 dB HL). The pure tone average of the better ear (Table 1) was also similar in all 3 groups. Groups were also comparable on their age (*F*[2,57] = 0.93, *P* = 0.40), nonverbal IQ (as assessed by performance on the WASI Matrix Reasoning subtest, *F*[2,57] = 0.87, *P* = 0.42), and working memory (calculated using the forward and backward digit span subtests of the WASI, *F*[2,57] = 0.54, *P* = 0.59).

Participants completed a set of tests that assessed their perceptual and musical abilities. Specifically, frequency discrimination and duration discrimination thresholds were determined using an adaptive staircase procedure (as described in Boebinger et al. 2015; implemented in MATLAB toolbox MLP (Grassi and Soranzo 2009)). Although guitarists had lower thresholds than beatboxers and nonmusicians on the frequency measure, no differences were observed when comparing beatboxers and nonmusicians (Table 1 shows the mean and standard deviation on each of these measures, Supplementary Table S7 has relevant statistics). No group differences were observed for duration discrimination. Participants also completed the rhythm judgment (deciding whether two tunes differed in rhythm) and metric perception (judging whether a tune was a waltz or a march) from the Montreal Battery of Evaluation of Amusia (Peretz et al. 2003). On metric perception, only the guitarists had higher scores than beatboxers and nonmusicians. However, on the rhythm perception test, both beatboxers and guitarists outperformed nonmusicians (see Supplementary Table S7 for relevant statistics). Additionally, all participants completed a self-report measure of musical sophistication, the Goldsmiths Musical Sophistication Index (Müllensiefen et al. 2014). Here, the beatboxers rated themselves similarly to the guitarists on all indices besides formal training, and their general musical sophistication scores were significantly higher than those of nonmusicians (Table 1, also see Supplementary Table S7).

### Stimuli

The guitar and beatboxing clips used were novel pieces, and were unfamiliar to participants. Beatbox pieces were recorded by a professional beatboxer (H.Y., known professionally as Reeps One) in an anechoic chamber. A professional guitarist created the guitar pieces in a studio setting. Both musicians created pieces that were both technically challenging and aimed at showcasing a range of styles. These pieces were edited in Audacity to create clips of durations between 3 and 5 seconds. The intensity of the clips was root mean square normalized to the same level and they were presented at a comfortable listening volume.

### MRI Acquisition

All MRI data were acquired on a 1.5 T Siemens Avanto scanner with a 32-channel receive-only head coil. Functional MRI images were acquired using a T2*-weighted gradient-echo planar imaging sequence, which notionally covered the whole brain (repetition time: 9.5 s, acquisition time: 3.4 s, echo time: 50 ms, flip angle: 90°, field of view: 192 × 224). In total, 40 axial slices with a thickness of 2 mm and an interslice gap of 1 mm were acquired in ascending order. These slices notionally give whole-brain coverage, but in participants where this was not possible we aimed to cover frontal, temporal, and inferior parietal regions, and as much of cerebellum as possible. A sparse acquisition design was used to present the stimuli in silence (Hall et al. 1999). Stimuli were presented in a 6.1 s silent period, which was followed by 3.4 s of image acquisition.

Two runs of the listening task were acquired. Each run comprised 32 trials of each condition (beatboxing/guitar music) interspersed with a resting baseline (32 instances of rest) presented in a pseudorandomized order (the order differed for each participant). The randomization was constrained so an instance of each category (beatbox music, guitar music, and rest) occurred in triads, ensuring that instances of each category were never spaced more than 5 trials apart. The onsets of the musical stimuli were jittered between 0 and 0.5 s. The same musical pieces were repeated for the second run in a different order. Participants listened to each of the musical pieces via in-ear Sensimetric earphones (http://www.sens.com/products/model-s14/), they were not asked to perform any task as they listened. Participants were specifically asked not to move their mouths or hands and to keep their eyes open. Cameras positioned over the face and dominant hand of the participant were used to assess compliance with instructions; all participants followed these instructions.

Interspersed between these two musical listening runs, participants completed a run where they listened to unrelated sounds. The results of this run are not reported in this article. Following the listening runs, participant also completed a mouth and hand localizer, where they had to move their hands or mouths in a sequence of actions in response to visual prompts. In the hand condition, participants performed bimanual sequential actions, touching each of their fingers to the thumb and then making a fist. In the mouth condition, participants alternated between pursing the lips and touching the tip of their tongue to the roof of their mouth. For this run, scanning was continuous (repetition time: 3.4 s, echo time: 50 ms, flip angle: 90°, field of view: 192 × 224). The order of events was optimized using optseq2 (https://surfer.nmr.mgh.harvard.edu/optseq). In addition to this localizer, participants also completed a phonation versus breathing localizer (data not described here). None of the listening/localiser tasks were described as the primary task. Instead, participants were simply informed that they would be listening to sounds in the scanner, including music and these hand/mouth sounds, and would be asked to perform specific hand/mouth actions when they received visual prompts (which they practiced outside of the scanner).

Finally, a T1-weighted structural scan was also acquired for registration purposes from all participants (resolution 1 × 1 × 1 mm^3^, repetition time 2730 ms, echo time 3.57 ms, flip angle 7°).

### Univariate Analyses

Data were analyzed using Statistical Parametric Mapping (SPM8). Scans were realigned, unwarped, and spatially normalized to 2 mm^3^ isotropic voxels using the parameters derived from the segmentation of each participant’s T1-weighted image, and smoothed with a Gaussian kernel of 8-mm full-width at half maximum.

The 2 stimulus conditions (and 6 movement regressors of no interest) were entered into a general linear model at the first level. The canonical hemodynamic response function was used to model the onsets and durations of the 2 musical conditions. The “rest” condition provided an implicit baseline. Similar to Wild, Davis, Johnsrude (2012), we did not correct for serial autocorrelation due to our long TR. Furthermore, no high-pass filter was applied to the data. The microtime resolution was set at 18 and the onset time was set to 100. For each subject, we generated a (beatbox music > rest) and a (guitar music > rest) contrast at the first level. These contrast images were used at the second level in one-sample *t*-tests to characterize areas where all participants showed greater activity for (guitar music > rest) and (beatbox music > rest).

For each subject, we also generated a [beatbox > guitar] contrast image at the first level. These contrast images were used at the second level to conduct statistical tests using the partitioned error term approach. One sample *t*-tests for [beatboxing > guitar] analyzed with an *F* contrast were used to characterize areas showing a relative increase in activity to beatboxing or guitar respectively within each group (see Fig. 2*A*, where [beatbox > guitar] activity is shown in red–yellow; and [guitar > beatbox] activity is shown in blue–light blue). To assess the group × condition interaction we conducted a one-way ANOVA testing the effect of group (guitarist/beatboxer/nonmusician) with an *F* contrast. In this ANOVA, the factor “group” was specified as being an independent measure, with unequal variance. To understand which differences were driving the group × condition interaction at the whole brain level, the simple effects of beatboxing > guitar for each group difference (e.g., guitarists vs. beatboxers, guitarists vs. nonmusicians, beatboxers vs. nonmusicians) were assessed with independent samples *t*-tests for each group difference inclusively masking within areas that showed the group × condition interaction. Unless otherwise specified, all statistical maps are thresholded at a peak level of *P* < 0.05 (family-wise-error or FWE corrected at the voxelwise level).

For region-of-interest (ROI) analyses focusing on hand and mouth regions, we used 2 separate one-sample *t*-tests, entering the contrast of [hand > rest] and [mouth > rest], respectively, from the hand/mouth localiser for all participants. From these analyses, peak co-ordinates in sensorimotor cortex were obtained that denoted left hand area [−36 –28 50], right hand area [38 –28 48], left mouth area [−52 –10 40], and right mouth area [56 –8 40]. Using the SPM toolbox marsbar, we created 10-mm spheres centered on these peaks and extracted mean beta values for the [beatbox > guitar music] contrast for each participant. Using SPSS, we conducted a 2 × 2 × 3 ANOVA on the mean beta values to determine if hemisphere (left/right), region (mouth/hand), and group (beatboxers/guitarists/nonmusicians) modulated beatbox > guitar activity within these ROIs.

### Multivariate Analyses

ICA or Independent Component Analysis is a multivariate analysis technique that can extract information from the data that is not always apparent from a subtractive univariate analysis (Geranmayeh et al. 2014). This approach takes advantage of fluctuations in fMRI data to separate it into maximally independent spatial components, which explain unique variance in the data. Each component is associated with a timecourse, which can be related to the task, artifacts related to movement or blood flow, or both.

Here, a group concatenation ICA was carried out using Probabilistic ICA (Beckmann and Smith 2004) as implemented in MELODIC Version 3.14, part of FSL. In this approach, the data from all subjects is temporally concatenated, with data from one subject following the other. We included data from all 60 participants in this analysis, so that we could derive an unbiased set of networks that would represent our 3 groups equally well. MELODIC does not have any information about the number of datasets that are provided, or where the separation is between subjects or runs. The following data preprocessing was applied to the input data: manual denoising using an ICA at the single-subject level to remove artifactual components, registration of this data to standard space, masking of nonbrain voxels; voxelwise demeaning of the data; and normalization of the voxelwise variance. When performing ICAs at the single-subject level, in each participant, components were marked as signal or noise with reference to the hand-classification scheme proposed by Griffanti et al. (2017). Components characterized as signal were biologically plausible and in gray matter, had relatively smooth time series, and were characterized by power in the low frequencies. Those characterized as noise included maps commonly associated with motion (for instance, a ring around the brain), those that closely followed vasculature, or those that appeared nonbiological with a large number of small, unrelated clusters. If we were unsure about whether a component represented noise or signal, it was marked as signal so that it was retained for further analysis. Preprocessed data were whitened and projected into a 13-dimensional subspace using probabilistic Principal Component Analysis. The number of dimensions was automatically estimated using the Laplace approximation to the Bayesian evidence of the model order (Minka 2000; Beckmann and Smith 2004). The whitened observations were decomposed into sets of vectors which describe signal variation across the temporal domain (time-courses), the session/subject domain and across the spatial domain (maps) by optimizing for non-Gaussian spatial source distributions using a fixed-point iteration technique (Hyvarinen 1999). Estimated component maps were divided by the standard deviation of the residual noise and thresholded by fitting a mixture model to the histogram of intensity values (Beckmann and Smith 2004).

We then evaluated the group spatial components, which are the output of the MELODIC analysis. Of the 13 components, one was clearly related to noise and was not analyzed further. Although the 12 remaining components did overlap spatially, pairwise spatial correlations between these maps (Supplementary Fig. S3) did not exceed *r* = 0.13. We also spatially correlated these 12 components with a reference set of resting state networks (Smith et al. 2009) to identify domain-general attentional and cognitive control networks at the group level.

Dual regression involves regressing the individual subject fMRI datasets against the group component spatial maps, the first output is subject-specific time-courses. As we had 2 runs for each participant, we used dual regression to extract run-specific time courses for each subject for each group spatial component. In a typical dual regression, the next step would be to regress individual subject fMRI datasets against the subject-specific time courses to obtain subject-specific component spatial maps. However, while conducting a task-based ICA the main issue is how well subject-specific component time courses relate to the applied design matrix. Consequently, we used the tool fsl_glm to regress the subject-specific time courses against the design matrix for the task. This was done separately for each run the participant completed. This allows us to estimate the contrast of parameter estimate (COPE) for our contrasts of interest (here, beatboxing vs. rest, guitar music vs. rest, beatboxing vs. guitar music) for each network. The COPEs for each contrast were then averaged over the 2 runs for each participant (i.e., [beatboxing > rest] for run 1 and run 2). We could then test COPE values across participants to identify components where activity was greater during [Listening > Rest] or components where a group × condition interaction was observed. These statistical analyses were conducted in SPSS v25.0. Given that ICA is a data-driven, model-free approach, it is appropriate to correct for multiple comparisons at this stage of the analysis, resulting in a Bonferroni correction for 12 components.

### Structural Analyses

To examine local changes in gray matter volume, structural data were analyzed with FSL-VBM (Douaud et al. 2007). First, structural images were brain-extracted and gray matter-segmented before being registered to the MNI-152 standard space using nonlinear registration. The resulting images were averaged and flipped along the *x*-axis to create a left-right symmetric, study-specific gray matter template. Second, all native gray matter images were nonlinearly registered to this study-specific template and “modulated” to correct for local expansion (or contraction) due to the nonlinear component of the spatial transformation. The modulated gray matter images were then smoothed with an isotropic Gaussian kernel with a sigma of 3 mm (~7 mm FHWM). Finally, permutation-based nonparametric testing (5000 permutations) was applied within the framework of the general linear model. Contrasts examined in this analysis were [musicians > nonmusicians], [guitarists > nonmusicians], [beatboxers > nonmusicians], [guitarists > beatboxers] and vise versa, while covarying out effects of gender, age, and nonverbal IQ. Results were considered significant for *P* < 0.05, corrected for multiple comparisons using threshold-free cluster enhancement (tfce), which avoids using an arbitrary threshold for initial cluster formation.

## Results

**Figure 1.**
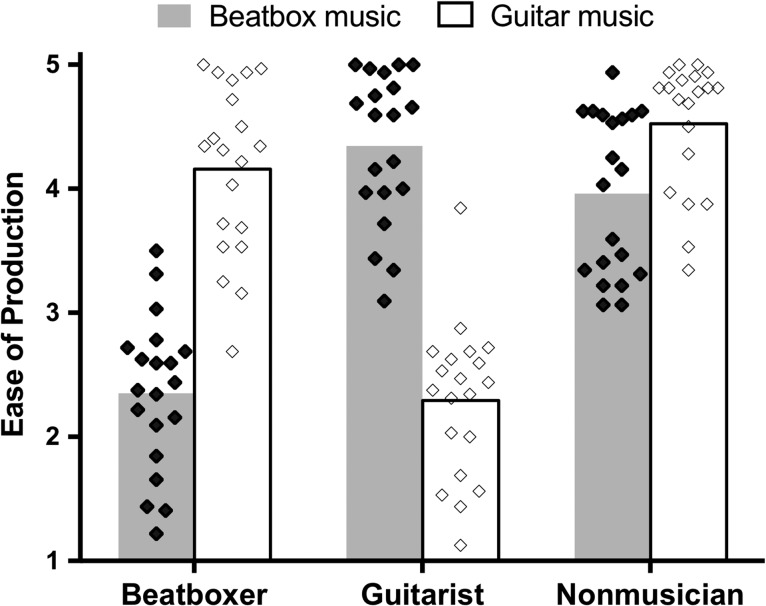
Postscan behavioral ratings provided by each group when listening to clips from the two music conditions (beatbox—gray bars/filled diamonds; guitar—clear/unfilled diamonds). Each data point represents a participant (*N* = 20 in each group), the bars depict the mean in both conditions. Lower ratings indicate that the stimuli are perceived to be easier to produce.

We scanned 20 guitarists, 20 beatboxers, and 20 nonmusicians as they listened to 3–5 s excerpts of novel guitar and beatbox music that were produced by experts. These groups were matched for age, basic cognitive, and hearing abilities (see Table 1 for demographic details). At the end of scanning, all participants rated the pieces of music they heard with respect to ease of production (Fig. 1). Participants were not told about these ratings in advance of the scan, but were asked to do them after they completed the scans (along with other components of the behavioral battery). For these behavioral ratings, we observed a Group (nonmusician/beatboxer/guitarist) × Condition (guitar/beatbox pieces) interaction, *F*(2,57) = 119.3, *P* < 0.001. Post hoc *t*-tests showed that guitarists rated the guitar stimuli (*M* = 2.3, SD = 0.6) as easier to produce than beatboxing (*M* = 4.3, SD = 0.6), *t*[19] = 10.3, *P* < 0.001, whereas beatboxers showed found beatboxing (*M* = 2.4, SD = 0.6) easier to produce than guitar music (*M* = 4.2, SD = 0.7), *t*[19] = 9.0, *P* < 0.001. We also found that nonmusicians rated the beatboxing pieces (*M* = 4.0, SD = 0.7) as easier to produce than the guitar music (*M* = 4.5, SD = 0.5), *t*[19] = 4.2, *P* < 0.001. This may be because they perceived a vocal stimulus to be easier to simulate than an instrumental one.

For both musical conditions relative to a resting baseline, increases in activity were observed in primary auditory cortex and extending into superior temporal gyrus, as well as in the brainstem and the cerebellum (Supplementary Table S1 shows peak activation for listening to each music condition in the 3 groups). We did not further examine any differences in the 2 musical conditions relative to rest, as we were interested in how each group differentially responded to the 2 musical conditions rather than to listening to music more generally.

**Figure 2.**
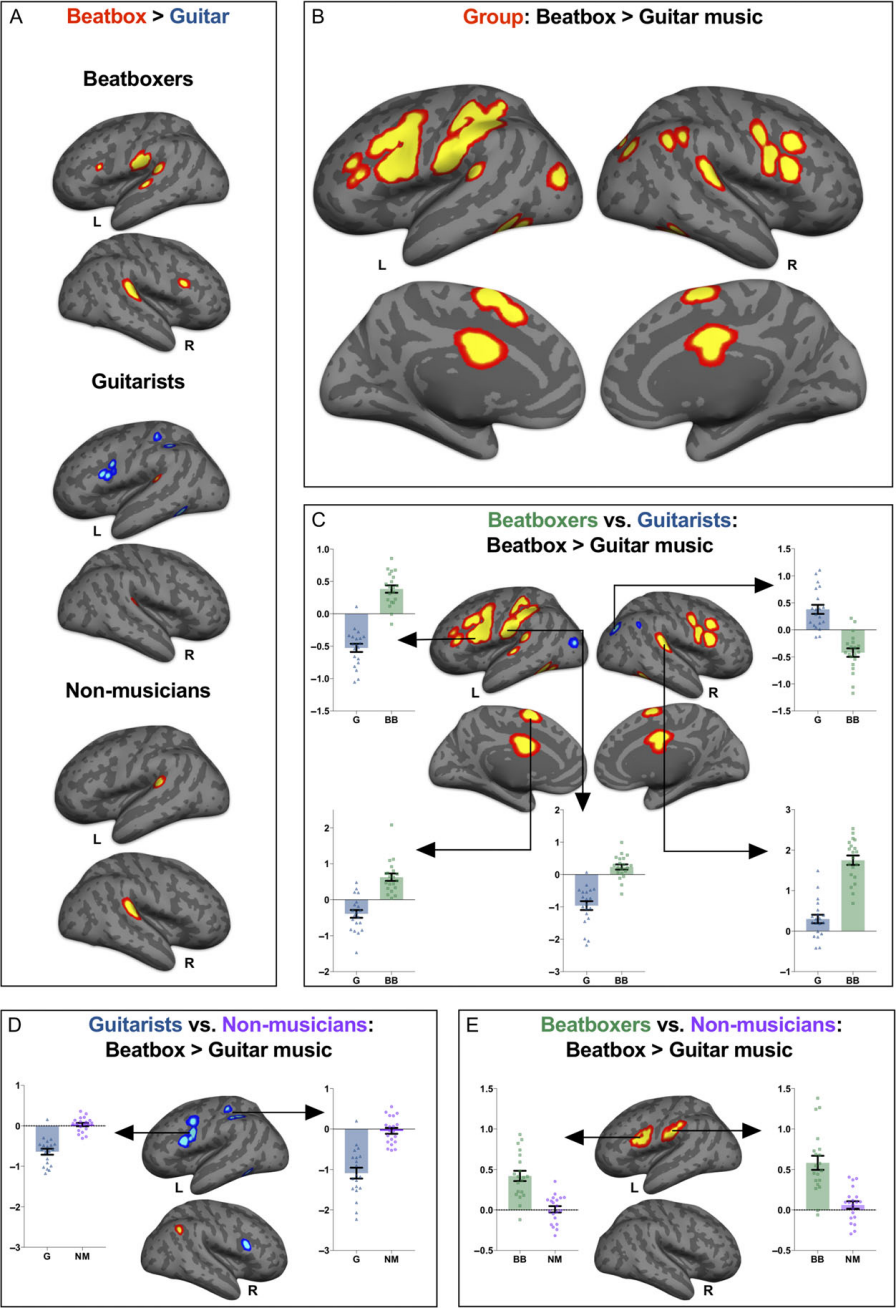
Depicts the results of univariate analyses conducted in SPM to explore group × condition modulation in listening activity. Panel (*A*) shows activity for the beatbox > guitar music contrast in beatboxers, guitarists, and nonmusicians. Regions where activity for listening to beatboxing exceeds that of listening to guitar music are shown in red–yellow, regions where activity for guitar music exceeds that of guitar music are shown in blue–light blue. This highlights that beatboxers and guitarists show increases in activity over dorsal stream regions for music they can produce. Nonmusicians do not show a modulation by condition in these dorsal stream regions, but do show increased activity for beatbox > guitar music in superior temporal cortex bilaterally. Note that analyses were inclusively masked by regions that showed the group × condition interaction, which are shown in panel (*B*). Panel (*B*) depicts regions where beatbox > guitar activity is modulated by group membership (beatboxer/guitarist/nonmusician), highlighted in red/yellow. These include left and right inferior frontal cortex, left postcentral gyrus and inferior parietal regions, supplementary and cingulate motor areas, and inferior temporal cortex and posterior superior temporal gyri bilaterally. For panels (*C*–*E*), analyses were inclusively masked by the regions depicted in panel (*B*). Panel (*C*) shows regions where beatboxers and guitarists have a differential response to the beatbox > guitar music contrast. Areas highlighted in red–yellow show regions where there is positive instrument-specific modulation by musicians, and those in blue show negative instrument-specific modulation. Bar graphs show mean beta values from highlighted clusters, bars in blue show the mean activity for beatbox > guitar music in guitarists, the bars in green show the same for the beatboxers. Positive values indicate more activity for beatboxing, whereas negative values suggest more activity for guitar music. Error bars denote ±1 standard error of the mean (SEM). Each data point represents an individual participant. Panel (*D*) shows regions where guitarists and nonmusicians have a differential response to the beatbox > guitar music contrast. Bar graphs show mean beta values from highlighted clusters, bars in blue show the mean activity for beatbox > guitar music in guitarists, the bars in purple show the same for the nonmusicians. Error bars denote ±1 SEM. Each data point represents an individual participant. Panel (*E*) shows regions where beatboxers and nonmusicians have a differential response to the beatbox > guitar music contrast (*P* < 0.05 FWE cluster-corrected). Bar graphs show mean beta values from highlighted clusters, bars in green show the mean activity for beatbox > guitar music in beatboxers, the bars in purple show the same for the nonmusicians. Each data point represents an individual participant. Error bars denote ±1 SEM. Thresholded activation maps (*P* < 0.05 FWE) for each contrast described here are registered to and displayed on a cortical surface using Freesurfer. The cortical surface was generated using the average T1 of the MNI-152 template. Activity in the cerebellum is not shown.

Our first objective was to examine whether any regions were sensitive to a Group (nonmusician/beatboxer/guitarist) × Music condition (guitar/beatbox) interaction. We found a set of regions sensitive to this interaction (Supplementary Table S2 and Fig. 2*B*), including bilateral inferior frontal cortex, left inferior parietal cortex, left inferior temporal cortex, lobule VI/VII in both cerebellar hemispheres and in supplementary motor area bilaterally (at *P* < 0.05 family-wise error [FWE] corrected at peak voxel level).

One-sample *t*-tests were used to establish the pattern of beatbox > guitar activity in each group (see Fig. 2*A* and Supplementary Table S3). We restricted our analyses to the set of regions that showed a significant group × condition interaction, by applying an inclusive mask during analysis. Beatboxers showed greater activity for [beatbox > guitar music] in the superior temporal gyri, cerebellum (lobule VI), the supplementary motor area, and in the inferior frontal gyrus bilaterally, as well as the left precentral gyrus. Only one region, the right middle occipital gyrus, showed greater activity for [guitar > beatbox music]. Guitarists also showed increased activity in bilateral superior temporal gyri for the contrast [beatbox > guitar music]. However, in the left inferior parietal cortex, left inferior frontal cortex, the left inferior temporal cortex, and in the supplementary motor area, guitarists exhibited greater activity for [guitar > beatbox music]. In contrast to beatboxers, who engaged dorsal stream regions when listening to beatboxing, guitarists recruited these regions when listening to guitar music. Nonmusicians did not differentially activate dorsal stream regions for either beatboxing or guitar music (at a threshold of *P* < 0.05 FWE). Again, for [beatbox > guitar music] in the nonmusicians, we observed increases in activity in bilateral superior temporal gyri. No regions were significantly activated by guitar music to a greater extent that beatbox music.

To interpret differences by listening condition across the 3 groups, we conducted 2-sample *t*-tests comparing the [beatbox > guitar music] contrast across guitarists and controls, beatboxers and controls, and beatboxers and guitarists, with analysis restricted to regions that showed the [group × condition] interaction described previously. The directionality of differences in these regions was determined by extracting mean beta values for the [beatbox > guitar] contrast from the cluster using the marsbar toolbox for SPM, which were further analyzed using SPSS. When brain responses of guitarists and nonmusicians for the [beatbox > guitar music] contrast were compared (Fig. 2*D* and Supplementary Table S4B), we observed differences across left and right inferior frontal cortex, left postcentral gyrus, left supplementary motor area, and left inferior temporal cortex. This reflected the fact that guitarists showed a preference for guitar music over beatboxing in these regions, whereas nonmusicians showed the opposite preference. However, in the right inferior parietal lobule, guitarists showed a stronger preference for beatboxing relative to guitar music, while nonmusicians showed no strong evidence of modulation. When comparing activity for the [beatbox > guitar music] contrast in beatboxers and nonmusicians (Fig. 2*E* and Supplementary Table S4A), at a voxelwise threshold of *P* < 0.05 FWE, we only observed a significant difference over the right cerebellum, where beatboxers showed a stronger beatbox > guitar modulation than nonmusicians. However, on reducing the statistical threshold to *P* < 0.05 FWE corrected at the cluster level, we observed a pattern of expertise-driven responses, with beatboxers showing greater activity for beatboxing over guitar music than nonmusicians did in left inferior frontal cortex and left inferior parietal cortex (Fig. 1*E*).

When we compared beatboxers and guitarists on the beatbox > guitar music contrast (*P* < 0.05 FWE voxelwise), group differences were observed in a range of dorsal stream regions such as inferior frontal cortex bilaterally, as well as in the left inferior parietal cortex (Fig. 2C, Supplementary Table S4C). We then assessed the directionality of differences in these clusters. In the left and right inferior frontal cortex, left and right cerebellum, left and right inferior temporal cortex, and in supplementary motor area, cross-over effects of expertise were observed with musicians showing greater activity for music they could produce (Fig. 1*C*). In the left inferior parietal lobe, guitarists exhibited a strong preference for guitar music, but the difference between the 2 musical types was less marked in the beatboxers. In the right and left middle occipital gyrus, as well as in the right inferior parietal lobe, guitarists had greater activity for beatbox > guitar music whereas beatboxers showed greater activity for guitar > beatbox music. These regions consequently showed decreases in activity for music musicians could play. Finally, both groups exhibited a [beatbox > guitar music] preference in the right superior temporal gyrus, but this was substantially larger in the beatboxers.

To examine if there were any expertise-general effects associated with musicianship we constructed the conjunction null of 1) [guitarists > nonmusicians] for [beatbox music > rest] and 2) [beatboxers > nonmusicians] for [guitar music > rest]. These contrasts were chosen to avoid including music that the musician played when looking for an expertise-general effect, as that would simply lead to us noting the increases in activity over dorsal stream region. We did not find any regions that showed expertise-general effects, even when using a threshold of *P* < 0.005 uncorrected for whole-brain comparisons.

**Figure 3.**
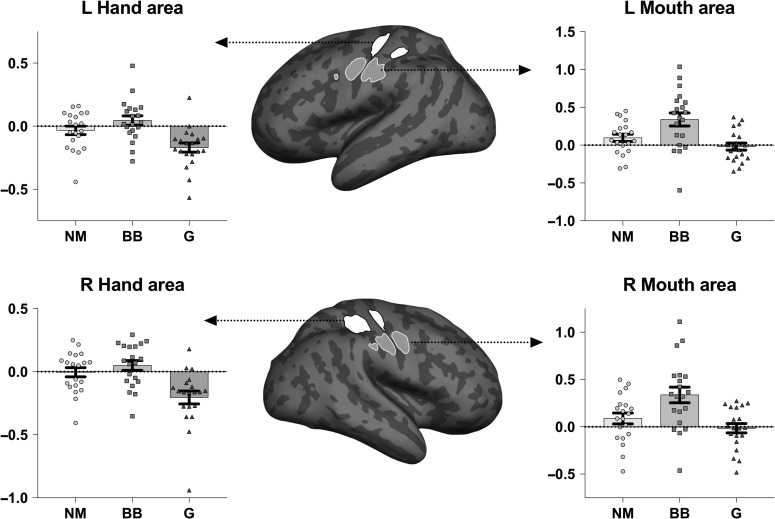
Depicts the results of univariate ROI analyses, where mean beta values for left and right hand and mouth regions were extracted for beatbox > guitar music in each participant. Positive values indicate more activity for beatboxing, whereas negative values suggest more activity for guitar music. Error bars depict ±1 standard error of the mean. Each data point represents an individual participant. Nonmusicians (NM) are represented by filled circles, beatboxers (BB) by filled squares and guitarists (G) using filled triangles. These graphs clearly show that guitarists, but not the other 2 groups, show greater activity for guitar music in hand regions. In the mouth regions, beatboxers, but not the other 2 groups, show greater activity for beatboxing. The specific ROIs we sampled from are highlighted in the figure, and were derived from group activity for hand and mouth movements.

Given reports of somatopic representations in listening (Gazzola et al. 2006), our next objective was to examine whether musicians showed any effector-specific action-perception couplings in hand and mouth regions specifically (Fig. 3). At the end of the listening scanning session, all participants completed a functional motor hand/mouth localizer so we could independently localize these sensorimotor regions in our participants (see Materials and Methods for details about how ROIs were constructed). We then assessed whether hemisphere (left/right), effector (hand/mouth region) and group (nonmusician, beatboxer, guitarist) modulated [beatbox > guitar activity] in these 4 ROIs (Fig. 3). We were interested in the interaction between effector region and group, as we expected signal to be different in different across brain regions. The interaction between effector region and group was significant, *F*(2,57) = 3.61, *P* = 0.033. A main effect of hemisphere was not observed, *F*(1,57) = 0.01, *P* = 0.93, and interactions of hemisphere with group and effector region were also not significant (*P* > 0.7). For follow-up analyses, we averaged activity over right and left hand areas, and right and left mouth areas. In hand areas, group membership had a significant effect on neural activity, *F*(2,57) = 11.07, *P* < 0.001. Post hoc Bonferroni-corrected comparisons showed this was driven by a significant difference in means between guitarists and nonmusicians, *P* = 0.005, as well guitarists and beatboxers, *P* < 0.001. An expertise-driven preference for guitar music relative to beatboxing was observed in the guitarists in hand areas. Beatboxers and nonmusicians showed no significant differences in beatbox > guitar activity in the hand areas, *P* = 0.602. Activity in mouth areas was also modulated by group, *F*(2,57) = 9.93, *P* < 0.001. This group difference was driven by beatboxers showing an increased preference for [beatbox > guitar music], relative to the guitarists (*P* < 0.001), and to the nonmusicians (*P* = 0.012). Guitarists and nonmusicians showed comparable activity for beatboxing and guitar music (*P* = 0.56). As an additional follow-up, we repeated these analyses using individualized hand and mouth ROIs instead of 10 mm spheres. This confirmed a preference for the music musicians can play, but this preference was more general, that is, it was observed in both mouth and hand areas, in guitarists and beatboxers (see Supplemental Data for details).

We then conducted data-driven ICA (see Materials and Methods for further details) to examine spatiotemporal network activity in musical perception, which allows us to describe 1) the spatiotemporal networks engaged in musical perception and 2) the effects of expertise on these networks. Using this multivariate ICA approach allows us to derive a set of spatial components and associated time courses for each participant. Our analyses indicated the presence of 13 spatially independent components (the number of components was automatically estimated to avoid bias), of which 12 could be considered non-noise (shown in Supplementary Fig. S1 and Supplementary Table S5). We spatially correlated these networks with a set of reference networks which are described in terms of their functional relevance (Smith et al. 2009) to derive appropriate labels for them (Supplementary Fig. S1). If there were no strong correlations with the reference set, we use anatomy to describe the network (C12). Typical caveats about reverse inference hold for any approaches which involve inferring cognitive function from brain activity (Poldrack 2011). However, this approach does allow us test some claims about underlying mental ontogeny, for example, examining whether “sensorimotor” or “executive” influences on frontoparietal regions are separable. Additionally, given the task-based nature of the design we can at least validate some network functions, such as whether the auditory network is responsive to listening.

**Figure 4.**
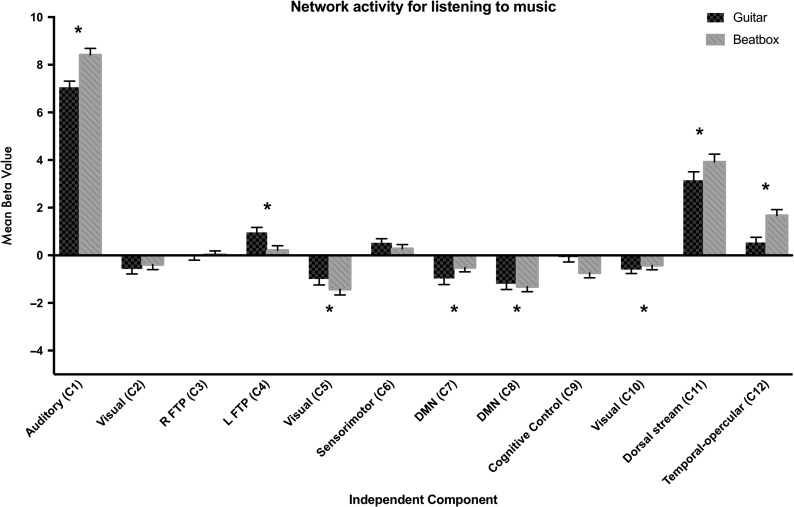
Depicts activity in for listening to guitar music (black) and listening to beatboxing (gray) in the 12 non-noise independent components. Networks were derived using a group concatenation approach implemented in FSL MELODIC, and dual regression was used to calculate cope values for each network in each participant for the contrasts beatbox > rest and guitar music > rest. Networks are labeled with a number as well as a functional descriptor based on their spatial distribution (see Supplementary Fig. S1). Graphs show mean beta values for these 2 contrasts, and error bars depict ±1 SEM. Networks C1 (auditory), C11 (higher-level sensorimotor), C12 (temporal–opercular), and C4 (left frontotemporo-parietal) show increases in activity when listening to music, whereas networks C5 (visual), C7 (default mode network), C8 (default mode network), and C10 (visual) show decreases in activity when listening to music.

First, we ascertained which networks were modulated by our tasks and whether they showed task-positive or task-negative activity. At an uncorrected threshold of *P* < 0.05, 11 of the 12 networks showed significant effects of listening. Only C3, or the right frontotemporo-parietal network, was not modulated by listening, *P* = 0.937. On applying a Bonferroni correction for 12 comparisons (corresponding to an alpha level of *P* < 0.05/12 or *P* < 0.004), we observed that networks C1 (auditory), *t*(59) = 30.2, *P* < 0.001, C4 (left frontotemporo-parietal), *t*(59) = 3.3, *P* = 0.002, C11 (higher-level sensorimotor network), *t*(59) = 11.8, *P* < 0.001, and C12 (bilateral temporal–opercular), *t*(59) = 5.5, *P* < 0.001, showed significant increases in activity in the 2 listening conditions compared with rest. Networks C5 (lateral visual), *t*(59) = 5.2, *P* < 0.001, C7 (default mode network), *t*(59) = −3.8, *P* < 0.001, C8 (default mode network), *t*(59) = 6.1, *P* < 0.001, and C10 (occipital pole + lateral visual), *t*(59) = 3.2, *P* < 0.001, showed decreases in activity for the 2 listening conditions relative to rest (Fig. 4).

**Figure 5.**
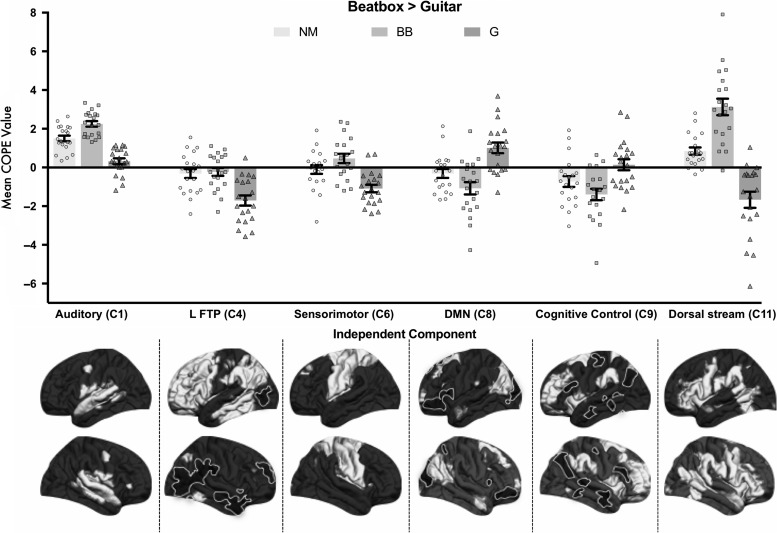
Demonstrates group × condition interactions in 6 of the 12 non-noise independent components derived using FSL MELODIC. The insets below each network label show regions included in the network, with regions that are positively covarying in white and those that show negative covariation in black. These are projected on the fsaverage cortical surface (for further details, such as activity on the medial surface, see Supplementary Fig. S1). Dual regression was used to derive subject-specific time courses for each of these 6 networks, and cope values for each network in each participant were calculated for the contrast beatbox > guitar music. In these graphs, values greater than 0 represent a preference for beatboxing relative to guitar music, whereas values less than 0 depict the opposite preference. Mean cope values for each group are shown by the bars, and the data points represent individual participants. Error bars depict ±1 SEM. Nonmusicians (NM) are depicted using filled circles, beatboxers (BB) with filled squares, and guitarists (G) using filled triangles. Musicians recruit auditory and sensorimotor networks as they listen to music they can produce, while inhibiting spatially overlapping default mode network (DMN) and executive control networks.

Next, we probed whether activity in any of our 12 networks was sensitive to the interaction between group × condition. Bonferroni-corrected analyses (*P* < 0.004, or *P* < 0.05 corrected for 12 comparisons) indicated that 6 of the 12 networks were modulated by the interaction of group and condition (Fig. 5). Of these, task-positive networks C6 (sensorimotor), *F*(2,57) = 12.8, *P* < 0.001 and C11 (higher-level sensorimotor network), *F*(2,57) = 43.9, *P* < 0.001, showed clear expertise-specific modulations. Task positive network C1 (auditory), *F*(2,57) = 43.4, *P* < 0.001, also showed modulation by expertise, but this effect was modulated by a main effect of condition on network activity. Task-positive network C4 (left frontotemporo-parietal), *F*(2,57) = 12.5, *P* < 0.001, showed a guitarist-specific expertise effect. Task-negative networks C8 (default mode network), *F*(2,57) = 14.5, *P* < 0.001 and C9 (executive control), *F*(2,57) = 7.2, *P* = 0.002, also showed expertise and effector-specific modulations. Below, we break down these group × condition interactions further (see Supplementary Table S6 for further details).

We first examined the 4 task-positive networks showing effects of expertise (Fig. 5). In network C6 (sensorimotor), beatboxers showed greater activity for beatboxing > guitar music. They significantly differed from guitarists, *t*(38) = 5.1, *P* < 0.001, who showed greater activity for guitar > beatbox music in this network. Nonmusicians did not show a strong modulation by listening condition in this network. There was a weak trend for beatboxers to differ from nonmusicians in their beatbox > guitar activity, *t*(38) = 1.7, *P* = 0.089. Guitarists and nonmusicians significantly differed from one another with respect to their activity for beatbox vs. guitar music in this network, *t*(38) = 3.3, *P* = 0.002. In network C11 (higher-level sensorimotor), a similar pattern of results was observed. Beatboxers showed greater activity for [beatbox > guitar music]. Their activity was significantly different to that seen in guitarists, *t*(38) = 8.0, *P* < 0.001, who showed greater activity for guitar music > beatboxing. The nonmusicians did not show a strong modulation by condition, and were significantly different from guitarists, *t*(38) = 5.5, *P* < 0.001, and beatboxers *t*(38) = 4.9, *P* < 0.001. In network C1 (auditory), the pattern of activity indicates that there was expertise-specific modulation, which was mediated by an auditory bias, with greater activity in the [beatbox > guitar music] condition seen in nonmusicians. This auditory bias may be driven by the fact that beatboxing is a vocal signal, containing information about factors such as the identity of the speaker and emotional state. The nonmusician preference for [beatbox > guitar music] was stronger than that of guitarists, *t*(38) = 5.6, *P* < 0.001. Guitarists did not show a strong bias for beatboxing, and activated this network equally for guitar music and beatboxing. Beatboxers showed stronger activity for [beatbox > guitar music] in this network relative to guitarists, *t*(38) = 9.2, *P* < 0.001, and nonmusicians, *t*(38) = 3.7, *P* = 0.001. Therefore, in networks C1, C6, and C11, musical experts showed significantly greater activity in the condition where they could produce the music. In task-positive network C4 (left frontotemporo-parietal), only guitarists had greater activity for [guitar > beatbox music], differing significantly from both beatboxers, *t*(38) = 4.4, *P* < 0.001, and nonmusicians, *t*(38) = 4.0, *P* < 0.001. Here, only guitarists showed significantly greater activity for the musical condition where they have experience producing music.

We then broke down the group × condition interactions in task-negative networks C8 and C9 (Fig. 5), where participants showed more activity during rest rather for listening to music (Fig. 4). In network C8 (default mode network), guitarists showed greater activity for [beatbox > guitar music], whereas beatboxers showed greater activity for [guitar music > beatboxing], *t*(38) = 4.9, *P* < 0.001. Nonmusicians did not show this modulation. Nonmusicians differed significantly from guitarists, *t*(38) = 3.7, *P* = 0.001, and showed a trend for a difference from beatboxers, *t*(38) = 2.0, *P* = 0.058. Network C9 (executive control) showed a similar inhibitory pattern. Significant differences were seen between guitarists and beatboxers, *t*(38) = 3.7, *P* < 0.001, guitarists and nonmusicians, *t*(38) = 2.2, *P* = 0.036, but not between beatboxers and nonmusicians, *t*(38) = 1.6, *P* = 0.11, for the [beatbox > guitar music] contrast. This indicated that musicians had significantly more inhibitory activity when listening to music they could produce. Taken together, these multivariate analyses reveal that specific effects of musical expertise are reflected in the engagement of domain-general attention/executive control networks and auditory-motor systems.

The set of regions where we observed expertise-related functional differences resemble those where a previous VBM study found gray matter differences in classical musicians (Gaser and Schlaug 2003). We consequently ran a VBM analysis to examine local changes in gray matter. However, we did not find any structural differences that survived *P* < 0.05 across groups (whole-brain-corrected for multiple comparisons using threshold free cluster enhancement, note that one-tailed differences in the beatboxer or guitarist > nonmusician contrasts exceed a *P* of 0.80), suggesting that local changes in gray matter are an unlikely explanation for the functional differences we obtain in sensorimotor regions. Structural differences in classical musicians are pronounced when musical training is started before 7 years of age (Steele et al. 2013; Vaquero et al. 2016). The later age of onset of training for nonclassical musicians (Table 1) might explain the lack of structural differences in these groups.

## Discussion

Neural systems recruited during the perception of music are modulated by previous sensorimotor experience. These results are consistent with findings suggesting that motor regions in the dorsal stream are recruited for listening to music (Grahn and Brett 2007; Chen et al. 2008b). However, we demonstrate that this increased sensorimotor activity is seen in an instrument-specific manner, with expert guitar players responding selectively to novel guitar sequences, and beatboxers responding selectively to novel sequences of beatboxing. This is the first time an instrumental-expertise specific neural effect within musicians has been established in nonclassical musicians. Expertise-related increases in activity are seen in the inferior frontal cortex and presupplementary/supplementary motor area bilaterally, as well as in left inferior temporal gyrus and inferior parietal cortex. By using network-based analyses, we further demonstrate that musical training is associated with expertise-specific recruitment of auditory and sensorimotor networks, and inhibition of domain-general default mode and executive control networks. Therefore, these results are a powerful demonstration of the idea that auditory perception is not simply driven by the properties of an auditory stimulus, but strongly influenced by the auditory-motor knowledge and experience that the listener brings to perception.

The sensorimotor regions differentially recruited by musicians have been found to play a role in higher-level motor control and co-ordination. Regions in inferior frontal cortex are involved in creating motor plans that are then executed by motor cortex, whereas those in inferior parietal cortex have a role in predicting the sensory consequences of movement (Dhanjal et al. 2008b). Recently, we have argued that the area at the boundary between the presupplementary motor area and supplementary motor area support flexible engagement of sensorimotor processes to guide auditory perception (Lima et al. 2016). Our present results provide further support to this argument, suggesting that activity in these regions is modulated by experience in a specific manner. Finally, we did also see an instrument-specific recruitment of left inferior temporal cortex during perception, which we had not anticipated. This region is typically engaged by language tasks that involve complex audiovisual links, such as picture naming (Krishnan et al. 2015) and reading (Price and Devlin 2003; Price 2012). This suggests that this region may be performing complex retrieval of audiovisual associations, such as the gestures associated with musical production.

An important question is whether our results might reflect sensorimotor engagement and expectancies, or a perceptual bias to the type of music that the musicians are familiar with. It is noteworthy that we did not observe suprathreshold instrument-specific effects over regions in the anteroventral stream, when comparing the 2 groups of musicians, or when comparing musicians to nonmusicians. This suggests that the most fundamental difference between these groups is not in how participants are engaging with the stimuli as a sound, that is, that musicians are not simply paying more attention to sounds they are more familiar with. It is rather suggestive, we argue, of a more specific mechanism—they might be engaging sensorimotor mechanisms more strongly when their previous experience provides them with the sensorimotor repertoire to do so. In addition, we had an a priori hypothesis about somatotopic increases in activity in primary motor and somatosensory regions as a consequence of sensorimotor experience. We therefore closely examined neural activity for perception in hand and mouth regions. We found that beatboxers recruited mouth areas when listening to beatboxing, and guitarists recruited hand areas when listening to guitar music. Our results therefore support effector-specific activity during listening, arguably driven by sensorimotor expectancies created by long-term experience producing and perceiving sound. Within a framework that ascribed no role to sensorimotor processing, it would be very difficult to explain why we would see this effector-specific difference.

A significant issue in the literature is whether effects of expertise are driven by sensorimotor or domain-general attentional factors, and this has been very difficult to address using standard univariate analyses. Areas that are thought to have sensorimotor functions and areas that play a role in executive control overlap significantly. Consequently, to address this issue, we used ICA to clarify the roles played by domain-general networks during music listening. ICA is particularly sensitive to the presence of domain-general networks, as it unmixes complex signals to reveal the presence of separable spatiotemporal activity (which can be canceled out in traditional univariate analysis) (Leech et al. 2012; Braga et al. 2013). Although previous work has examined functional connectivity during rest in musicians (Fauvel et al. 2014; Klein et al. 2015; Palomar-García et al. 2016), we have assessed task-level network level modulations. Similar independent component analyses have been conducted using EEG (Nolden et al. 2017), and a recent study used a ROI-based ICA approach with fMRI data (Burunat et al. 2017). However, here we go beyond simple musician versus nonmusician comparisons and capture whole brain network-level modulations in 3 different groups. With respect to expertise, we again did not find a domain-general advantage for musicians versus nonmusicians. Rather, we observed instrumental-expertise effects, some of which recapitulate findings from the univariate analyses. For example, we found that the set of regions we observed in the univariate analysis, including bilateral inferior frontal cortex, inferior parietal cortex, and supplementary motor areas were present within one separable network (C11). Interestingly, the spatial distribution of this network does not strongly correlate with any one single network from a reference set of networks determined from studies of adults resting in the scanner (Smith et al. 2009), indicating that it is not identical to previously established executive control, language, or motor networks. The reference networks are derived from healthy adults, unselected for any particular expertise. However, coactivation of this set of regions has also been observed in studies of visuomotor expertise (Calvo-Merino et al. 2005; Calvomerino et al. 2006), and is sometimes termed the action observation network (Cross et al. 2009). We therefore hypothesize that the coactivity of these regions might be driven by sensorimotor experience, and not domain-general factors. Somewhat surprisingly, the set of regions that become active in experts assessing their domain of expertise is almost identical across expert groups such as dancers and musicians, as well as within these groups (ballet dancers/capoeira dancers or beatboxers/guitarists) despite the different input to these brain regions and very different forms of training. In future studies, it would be worth exploring whether this network can be observed during rest in these expert groups, or whether the timecourse-coupling in these regions is driven by one’s evaluation of expertise.

However, our multivariate analysis also reveals the presence of other networks that are modulated by expertise, which are not obvious from the univariate analysis. For instance, as musicians listened to music played on instruments on which they have expertise, an auditory-motor network (C1) and a sensorimotor network (C6) were recruited. Additionally, musicians also inhibited domain-general networks while listening to music they could produce, such as a default mode (C8) and executive control networks (C9). This instrumental-specific modulation of attentional networks is notable, as it suggests that attentional networks respond specifically to the presence of familiar regularities in a musical style. Taken together, the univariate and multivariate results indicate that long-term sensorimotor experience has a distributed effect on both sensorimotor and domain-general brain networks, with the effect of expertise being broadly facilitatory for sensorimotor networks and inhibitory for domain-general ones. This would suggest that instrument-specific responses do not merely reflect increases in activity in auditory-motor networks, or changes in domain-general attention networks, but a complex combination of the two. Such overlapping changes are exceedingly difficult to pull out at a behavioral level, and our results suggest using a network level approach would greatly benefit our understanding of the interactions between domain-general and domain-specific systems. For instance, in future studies, it would be particularly intriguing to address how sensorimotor and attentional networks interact. One possibility is that the spontaneous engagement of dorsal stream regions would result in inhibition of attentional networks. On the other hand, it could be the case that domain-general networks respond to the presence of learned regularities in music one is familiar with, and this would then lead to the engagement of regions that those regularities are associated with. Network-level changes would also be interesting to explore in longitudinal studies of expertise, to examine when and which networks change over time.

Why might musicians recruit sensorimotor regions during perception? We tried to make our task as naturalistic as possible, by asking participants to listen to the music in the absence of any external task. This was done to limit any attentional or working memory demands, as well as limit motor activity due to motor priming. We also know that participants were not actively moving their hands or mouths as they listened. However, although our instructions to the 3 groups were identical, it is plausible that musicians could have been trying/or been unable to avoid recognizing or simulating aspects of the music they were listening to. Consequently, our interpretation of our sensorimotor activity is that internal motor models are automatically generated when participants listen to sounds they are experts at producing. This generation could be a consequence of long-term associations built between perception and production systems (Heyes 2010), and would reflect the richer sensorimotor representation for music in experts. Another possibility is that this activity might support working memory demands, which would be enhanced for music one can produce. Another explanation is that training allows the musicians to free themselves from inhibitory control, and use sensorimotor regions to make better predictions about what they are hearing (Pinho et al. 2014). Finally, we might argue that musicians deliberately try to simulate or learn about music they can produce, and it is this deliberate exertion that is reflected in the dorsal stream activity. Future studies are needed to pull apart these different explanations, and could involve assessing the behavioral and neural responses of these groups when different task demands are imposed, or by providing explicit directions to simulate or learn music. For example, using a dual-task paradigm, we could assess whether inhibiting the automatic engagement of these sensorimotor processes would lead to differences in auditory perception of music that musicians can produce.

A related issue is the functional relevance of sensorimotor activity, as a long-standing debate in the speech domain is whether sensorimotor activity during speech perception is epiphenomenal or necessary (for a recent review, see Skipper et al. 2017). Some authors take the middle ground, that is, sensorimotor activity during speech perception may be advantageous in certain situations, such as listening in noise (Davis and Johnsrude 2007). It is clear that top-down influences on perception, such as prior experience, allow listeners to make better predictions about ambiguous or unclear speech. However, in this case, we used a sparse-sampling design to limit noise, and it is difficult to imagine that musicians found the music they could produce more ambiguous to perceive than music they could not. However, there are studies suggesting that even when listening is easy (non-noisy situations), TMS to articulatory motor areas can disrupt perception (Mottonen et al. 2013), or change the excitability of motor cortex (Watkins et al. 2003; Panouillères et al. 2018). Skipper et al. (2017) suggest that the context provided by naturalistic speech perception tasks can increase the difficulty of the listening situation, and therefore lead to greater recruitment of sensorimotor regions. For example, adults showed greater activity in motor regions when listening to nonwords, relative to listening to words, perhaps reflecting the fact that they build sensorimotor models for words they have not encountered previously. Similarly, we believe participants might automatically simulate motor models for the style of music they had experience producing. There is some evidence that such internal simulation of movement typically makes aspects of perception, imitation, or learning easier. For example, Buccino and colleagues showed increases in activity in ventral premotor, inferior frontal and inferior parietal cortices as novices learnt to play guitar music (Buccino et al. 2004). Disrupting activity in the inferior frontal gyrus has been shown to impair covert imitation (Catmur et al. 2009). There is also behavioral work that supports the idea that internal simulation may improve aspects of perception, for instance, imitation of accented speech is known to improve intelligibility (Adank et al. 2010). Being able to covertly generate a motor model is associated with improved long-term auditory memory (Schulze et al. 2012). With respect to music, Keller (2012) has argued that internal simulation benefits action sequencing, co-ordination, timing accuracy, and motor force control during performance. We consequently hypothesize that the flexible engagement of these sensorimotor areas supports and shapes learning from the stimulus. The nature of our musical stimuli, which were novel and non-nameable, is therefore likely to have increased reliance on sensorimotor representations in nonmusicians. It is possible that these representations are less important when stimuli can be labeled. Future studies to explore the specific role of sensorimotor activity during perception could be done by making beatboxers and guitarists engage in specific articulatory suppression, or by using techniques like transcranial magnetic stimulation to target mouth/hand representations, while they perform perceptual tasks. This would allow us to assess the direct influence exerted by sensorimotor activity on musical listening behavior.

The presence of an expertise-specific neural effect in nonclassical musicians adds in important ways to previous evidence for functional specificity in studies that have tested classical musicians. For example, in a study where violinists were compared with actors, Dick et al. (2011) demonstrated that violinists were more likely to recruit premotor regions bilaterally, right inferior frontal cortex, and regions in the cerebellum when listening to violin excerpts relative to speech. In another study, a small sample of 9 violinists and 7 flutists showed increased activity for trained relative to untrained music in the precentral gyrus, inferior parietal cortex and supplementary motor area (Margulis et al. 2009). Our results are broadly consistent with these studies, but we demonstrate this effect for novel music in nonclassical musicians, who are largely self-taught. In addition, we demonstrate expertise-driven effector-specific modulation in hand/mouth areas during music perception, a question that has not been addressed by previous studies. Furthermore, in Dick et al. (2011) and in Margulis et al. (2009), comparisons are made within musicians, with no comparison to nonmusicians. This does not allow for the assessment of whether these effects are only observed in trained groups. Our results show that it is not musicianship generally, but instrument-specific experience, that modulates the response of dorsal stream regions to music. This strongly indicates that sensorimotor experience (and not the formal aspects of classical training such as ear training or musical theory) is the key factor in building these links.

In summary, our results establish that long-term sensorimotor experience relates to a stronger engagement of dorsal stream regions during perception, a finding that is particularly important to understanding how individual experiences might shape brain activity. We hypothesize this activation represents automatic activation of a unique kind of sensorimotor representation, one that is unavailable to those without the same sensorimotor experience. However, such representations are not crucial to perception, as nonmusicians are able to both perceive and make judgments about music. Rather, they may be particularly useful for imitation or learning. This is a fruitful direction for future studies to explore. Additionally, although our focus has been the cross-sectional comparison between nonclassical musicians, which was ideal as assess the correlates of long-term experience, an exciting avenue for future work is to implement a training/longitudinal design to firmly establish the causal direction of these effects. Our focus on dorsal stream regions being recruited by specific experience will also help reconcile diverse findings in the music literature, where some studies find motor activity for listening and others do not. Experience with stimulus type, and ability to generate sensorimotor expectations about the stimuli, might uniquely determine the presence of motor activity (Lima et al. 2016). An important implication of this finding for models of auditory processing is that it is not simply a task that modifies neural activity in dorsal stream regions, but also an individual’s prior expectations and experience that modifies the recruitment of dorsal stream regions. In addition to furthering our understanding of perception, our use of differently trained musician groups also allows us to evaluate the generalizability of learning that is a consequence of musical experience. Our findings indicate that different styles of musical expertise lead to distinct neural responses for the learned skill, however, enhanced responses occur within the same systems. This suggests that the neural effects of expertise are closely confined to learned behavior, but also that musical expertise broadly engages very similar areas. Future studies will be necessary to explore how the nature of representations in these regions differ for different musical styles, which is likely to have consequences for how we interpret the generalizability of different forms of musicianship.

## Supplementary Material

Supplementary Data

## Data Availability

Data and materials from all experiments are available from the corresponding author on request.
