# Supplementary material for: Beatboxers and Guitarists Engage Sensorimotor Regions Selectively When Listening to the Instruments They can Play
Source: Cereb Cortex. 2018 Aug 31;28(11):4063–79. doi: 10.1093/cercor/bhy208 (PMC6188551; doi:10.1093/cercor/bhy208)
Supplement: Supplementary Data [file bhy208_supplementarymaterial_18_08_02.docx]

**Supplementary Figure 1**

Panel A shows spatial maps obtained via an independent component analysis conducted on task data on an inflated brain, here, red-yellow colours denote voxels with positively correlated time-courses and blue denotes anti-correlated time-courses. Subcortical and cerebellar activity are not apparent here (see Supplementary Table 5 for a full set co-ordinates). Networks are grouped by putative function derived from correlations to reference resting state networks(Smith et al. 2009) further illustrated on Panel B. Panel C depicts the lack of spatial correlations between the task-derived independent components.


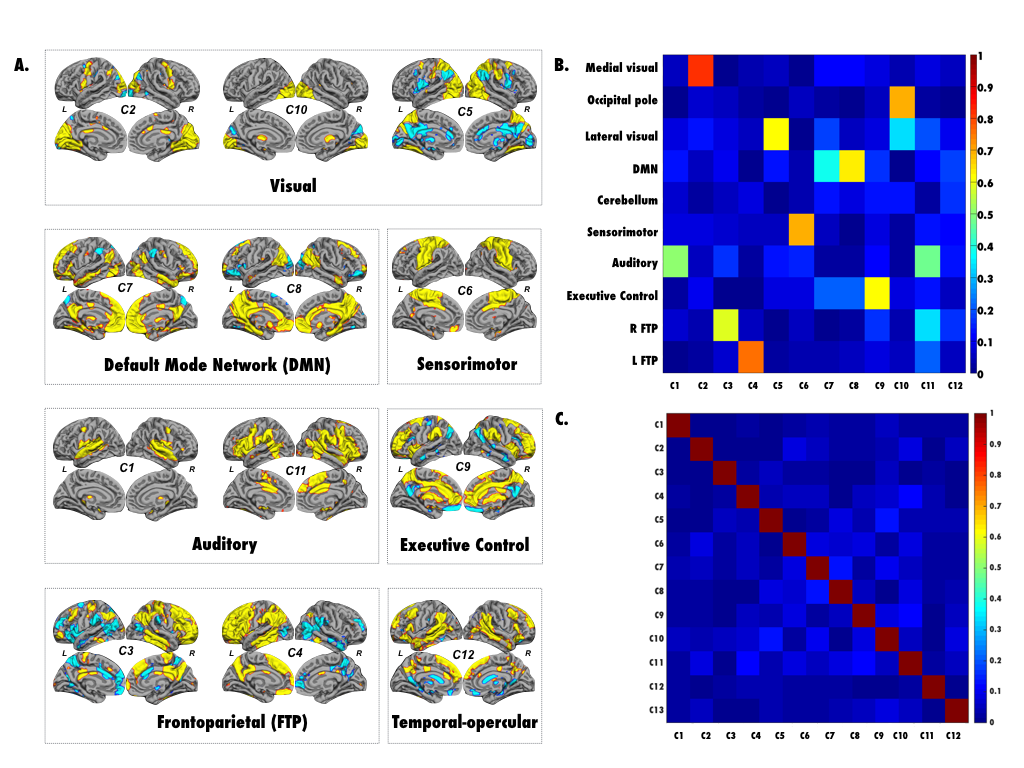


**Supplementary Table 1.** Peak co-ordinates that lie in gray matter for the main effects of listening to A. guitar music and B. beatboxing at p < 0.05, FWE-corrected at the whole brain level in the three groups.

| Area | X | Y | | Z | T_1,114_ | Z | P (FWE-corrected) | Extent |
| --- | --- | --- | --- | --- | --- | --- | --- | --- |
| *A. Guitar music > rest* | | | | | | | | |
| R Heschl’s gyrus/ superior temporal gyrus | **48** | | **-14** | **6** | 25.33 | >8 | <.001 | 21368 |
|  | **60** | | **-12** | **4** | 24.88 | >8 | <.001 |  |
|  | **-48** | | **-20** | **10** | 24.32 | >8 | <.001 |  |
| L Cerebellum (VI) | **-28** | | **-60** | **-24** | 12.55 | >8 | <.001 | 4196 |
|  | **-22** | | **-66** | **-26** | 11.12 | >8 | <.001 |  |
| L Cerebellum (VIIa) | **-42** | | **-64** | **-28** | 9.59 | 7.42 | <.001 |  |
| R midcingulate cortex | **2** | | **-2** | **34** | 9.43 | 7.33 | <.001 | 211 |
| R supplementary motor area | **4** | | **6** | **66** | 7.72 | 6.39 | <.001 | 458 |
| L inferior frontal gyrus (pars triangularis) | **-40** | | **38** | **2** | 7.11 | 6.02 | <.001 | 883 |
|  | **-48** | | **42** | **8** | 6.98 | 5.94 | <.001 |  |
|  | **-40** | | **32** | **8** | 6.90 | 5.89 | <.001 |  |
| L precentral gyrus | **-52** | | **-6** | **50** | 6.34 | 5.51 | 0.001 | 93 |
|  | **-50** | | **4** | **48** | 5.80 | 5.14 | 0.006 |  |
| R Cerebellum (I) | **10** | | **-36** | **-24** | 5.89 | 5.20 | 0.004 | 12 |
| R hippocampus | **24** | | **-14** | **-14** | 5.56 | 4.96 | 0.013 | 20 |
| L Calcarine Gyrus | **-2** | | **-92** | **-10** | 5.30 | 4.77 | 0.029 | 2 |
| L Middle Temporal Gyrus | **-56** | | **-52** | **10** | 5.23 | 4.72 | 0.037 | 6 |
| L insula | **-30** | | **18** | **2** | 5.22 | 4.71 | 0.038 | 3 |
| L supplementary motor area | **-4** | | **20** | **46** | 5.19 | 4.69 | 0.041 | 2 |
| R Cerebellum (I) | **18** | | **-36** | **-26** | 5.17 | 4.68 | 0.043 | 1 |
| L middle temporal gyrus | **-56** | | **-60** | **2** | 5.16 | 4.67 | 0.045 | 1 |
| L Cerebellum (Lobule I) | **-8** | | **-38** | **-26** | 5.16 | 4.67 | 0.046 | 1 |
| *B. Beatbox music > rest* | | | | | | | | |
| R superior temporal gyrus | **58** | **-16** | | **2** | 27.58 | >8 | <.001 | 14574 |
|  | **42** | **-20** | | **6** | 26.25 | >8 | <.001 |  |
|  | **48** | **-28** | | **8** | 23.22 | >8 | <.001 |  |
| L Heschl’s gyrus/ superior temporal gyrus | **-40** | **-22** | | **6** | 23.69 | >8 | <.001 | 11436 |
|  | **-38** | **-30** | | **14** | 20.34 | >8 | <.001 |  |
|  | **-58** | **-24** | | **10** | 20.14 | >8 | <.001 |  |
| L Cerebellum (VI) | **-28** | **-60** | | **-22** | 13.18 | >8 | <.001 | 5054 |
|  | **26** | **-60** | | **-24** | 11.51 | >8 | <.001 |  |
|  | **-20** | **-66** | | **-24** | 10.91 | >8 | <.001 |  |
| R supplementary motor area | **4** | **0** | | **68** | 10.94 | >8 | <.001 | 502 |
|  | **8** | **12** | | **72** | 7.44 | 6.22 | <.001 |  |
| L precentral gyrus | **-50** | **-4** | | **54** | 9.76 | 7.50 | <.001 | 398 |
| L midcingulate cortex | **0** | **-4** | | **36** | 8.18 | 6.66 | <.001 | 181 |
| L Cerebellum Lobule 1 | **-8** | **-38** | | **-26** | 6.66 | 5.73 | <.001 | 12 |
| R midorbital gyrus | **2** | **52** | | **-12** | 6.44 | 5.58 | 0.001 | 106 |

**Supplementary Table 2.** Peak co-ordinates in gray matter regions for the group x condition interaction, *p* < 0.05 FWE, when including guitarists, beatboxers and non-musicians.

| Area | X | Y | Z | F2,57 | Z | P (FWE-corrected) | Extent |
| --- | --- | --- | --- | --- | --- | --- | --- |
| L inferior frontal gyrus (pars opercularis) | **-50** | **10** | **24** | 60.44 | 7.67 | <.001 | 941 |
|  | **-48** | **8** | **32** | 41.21 | 6.73 | <.001 |  |
|  | **-50** | **8** | **14** | 39.46 | 6.62 | <.001 |  |
| L postcentral gyrus | **-52** | **-34** | **58** | 37.60 | 6.51 | <.001 | 1056 |
|  | **-62** | **-18** | **26** | 35.51 | 6.37 | <.001 |  |
| L Inferior Postcentral Lobule | **-46** | **-38** | **48** | 30.20 | 5.98 | <.001 |  |
| L Mid Cingulate Cortex | **0** | **0** | **34** | 34.87 | 6.32 | <.001 | 117 |
| Left Inferior Temporal Gyrus | **-50** | **-52** | **-16** | 33.58 | 6.23 | <.001 | 125 |
| R inferior frontal gyrus (pars triangularis) | **48** | **14** | **24** | 32.77 | 6.17 | <.001 | 348 |
| L supplementary motor area | **-4** | **4** | **62** | 31.02 | 6.04 | <.001 | 185 |
|  | **-6** | **16** | **50** | 21.49 | 5.18 | 0.007 |  |
| R Superior Temporal Gyrus | **66** | **-28** | **18** | 28.74 | 5.86 | <.001 | 132 |
|  | **60** | **-32** | **10** | 22.91 | 5.33 | 0.003 |  |
|  | **52** | **-32** | **12** | 18.33 | 4.82 | 0.035 |  |
| L Inferior Temporal Gyrus | **52** | **-48** | **-14** | 28.73 | 5.86 | <.001 | 31 |
| R Cerebellum (VI) | **24** | **-60** | **-24** | 28.66 | 5.85 | <.001 | 81 |
| L Cerebellum (VI) | **-22** | **-62** | **-24** | 26.26 | 5.64 | 0.001 | 49 |
| L Superior Temporal Gyrus | **-50** | **-38** | **18** | 24.33 | 5.47 | 0.002 | 33 |
| R precentral gyrus | **58** | **4** | **42** | 23.40 | 5.38 | 0.003 | 35 |
| L inferior frontal gyrus (pars triangularis) | **-46** | **36** | **16** | 21.28 | 5.16 | 0.008 | 27 |
| L insula | **-34** | **-4** | **12** | 20.95 | 5.12 | 0.009 | 7 |
| R Middle Occipital Gyrus | **36** | **-82** | **32** | 20.85 | 5.11 | 0.010 | 29 |
| R Inferior Postcentral Lobule | **60** | **-54** | **44** | 20.81 | 5.11 | 0.010 | 30 |
| L Superior Temporal Gyrus | **-64** | **-20** | **2** | 19.29 | 4.94 | 0.021 | 14 |
| R Middle Occipital Gyrus | **-40** | **-78** | **16** | 18.99 | 4.90 | 0.025 | 12 |
| L Middle Temporal Gyrus | **-52** | **-58** | **2** | 18.76 | 4.87 | 0.028 | 8 |
| R Middle Temporal Gyrus | **60** | **-52** | **4** | 18.50 | 4.84 | 0.032 | 3 |
| R Middle Frontal Gyrus | **50** | **8** | **52** | 18.34 | 4.82 | 0.035 | 4 |
| R Middle Occipital Gyrus | **40** | **-76** | **18** | 17.87 | 4.76 | 0.044 | 3 |
| L Frontal Operculum | **-24** | **30** | **-14** | 17.65 | 4.74 | 0.049 | 1 |

**Supplementary Table 3.** Peak co-ordinates in gray matter regions for the beatbox > guitar activity in A & B, beatboxers, C & D. Guitarists, E & F, *p* < 0.05 FWE.

| Area | X | Y | Z | T_19_ | Z | P (FWE-corrected) | Extent | |
| --- | --- | --- | --- | --- | --- | --- | --- | --- |
| *A. Beatbox > Guitar Music in Beatboxers* | | | | | | | | |
| R Superior Temporal Gyrus | **62** | **-32** | **16** | 17.07 | 7.21 | <.001 | | 131 |
|  | **52** | **-30** | **10** | 11.62 | 6.24 | <.001 | |  |
| L Superior Temporal Gyrus | **-50** | **-38** | **16** | 13.05 | 6.54 | <.001 | | 33 |
|  | **-62** | **-22** | **4** | 11.66 | 6.25 | <.001 | | 14 |
| L Postcentral Gyrus | **-66** | **-18** | **18** | 9.41 | 5.67 | 0.001 | | 59 |
| R cerebellum (VI) | **26** | **-58** | **-24** | 9.30 | 5.64 | 0.002 | | 26 |
| L cerebellum (VI) | **-26** | **-58** | **-26** | 8.62 | 5.44 | 0.004 | | 26 |
| R inferior frontal gyrus (pars triangularis) | **44** | **16** | **24** | 7.62 | 5.10 | 0.017 | | 15 |
| L supplementary motor area | **-2** | **0** | **66** | 7.43 | 5.03 | 0.023 | | 5 |
| L inferior frontal gyrus (pars triangularis) | **-52** | **16** | **28** | 7.36 | 5.00 | 0.026 | | 6 |
| *B. Guitar > Beatbox Music in Beatboxers* | | | | | | | | |
| R middle occipital gyrus | **44** | **-76** | **28** | 7.87 | 5.19 | 0.012 | 3 | |
| *C. Beatbox > Guitar Music in Guitarists* | | | | | | | | |
| R Superior Temporal Gyrus | **56** | **-30** | **10** | 11.02 | 6.10 | <.001 | 13 | |
| L Superior Temporal Gyrus | **-52** | **-36** | **16** | 7.18 | 4.93 | 0.034 | 1 | |
| R Inferior Parietal Lobule | **62** | **-54** | **42** | 6.99 | 4.86 | 0.046 | 1 | |
| *D. Guitar > Beatbox Music in Guitarists* | | | | | | | | |
| R Mid cingulate cortex | **2** | **-2** | **32** | 11.26 | 6.15 | 0.000 | 53 | |
| L inferior frontal gyrus (pars opercularis) | **-50** | **10** | **24** | 8.81 | 5.50 | 0.003 | 68 | |
|  | **-42** | **6** | **30** | 7.64 | 5.11 | 0.017 |  |  |
| L Inferior Temporal Gyrus | **-50** | **-56** | **-12** | 8.63 | 5.44 | 0.004 | 29 | |
| L inferior frontal gyrus (pars opercularis) | **-46** | **10** | **14** | 8.46 | 5.38 | 0.005 | 10 | |
| L Inferior Parietal Lobule | **-46** | **-38** | **52** | 7.83 | 5.17 | 0.013 | 58 | |
| L supplementary motor area | **-6** | **16** | **48** | 7.63 | 5.10 | 0.017 | 2 | |
| R Inferior Temporal Gyrus | **54** | **-50** | **-12** | 7.56 | 5.07 | 0.019 | 5 | |
| *E. Beatbox > Guitar Music in Non-musicians* | | | | | | | | |
| R Superior Temporal Gyrus | **52** | **-30** | **10** | 11.34 | 6.17 | <.001 | 124 | |
|  | **60** | **-32** | **8** | 11.02 | 6.10 | <.001 |  |  |
|  | **64** | **-28** | **20** | 10.17 | 5.89 | <.001 |  |  |
| L Superior Temporal Gyrus | **-48** | **-38** | **20** | 9.46 | 5.69 | 0.001 | 23 | |
|  | **-58** | **-38** | **18** | 7.61 | 5.10 | 0.021 |  |  |
| *F. Beatbox > Guitar Music in Non-musicians* | | | | | | | | |
| No suprathreshold clusters. | | | | | | | | |

**Supplementary Table 4A.** Peak co-ordinates in gray matter regions in a two sample T-test comparing beatbox > guitar music activity in beatboxers and controls, masked by regions that show a significant group x condition interaction. The voxels that are italicized are significant only at a p<0.05 cluster-corrected FWE

| Area | X | Y | Z | T_38_ | Z | P (FWE-corrected) | Extent |
| --- | --- | --- | --- | --- | --- | --- | --- |
| *A. Beatboxers > controls for beatbox > guitar* | | | | | | | |
| R cerebellum (VI) | **24** | **-58** | **-22** | 5.97 | 4.98 | 0.017 | 7 |
| *L inferior parietal lobule* | ***-68*** | ***-24*** | ***30*** | *5.18* | *4.47* | *0.011 (cluster)* | 265 |
| *L inferior frontal gyrus (pars opercularis)* | ***-50*** | ***8*** | ***12*** | *5.02* | *4.37* | *<.001 (cluster)* | 527 |
|  | ***-54*** | ***12*** | ***24*** | *4.83* | *4.24* |  |  |
| *B. Controls > Beatboxers for beatbox > guitar* | | | | | | | |
| No suprathreshold clusters | | | | | | | |

**Supplementary Table 4B.** Peak co-ordinates in gray matter regions in a two sample T-test comparing beatbox > guitar music activity in guitarists and controls, masked by regions that show a significant group x condition interaction.

| Area | X | Y | Z | T_38_ | Z | P (FWE-corrected) | Extent |
| --- | --- | --- | --- | --- | --- | --- | --- |
| *A. Guitarists > controls for beatbox > guitar* | | | | | | | |
| R inferior parietal lobule | **60** | **-54** | **44** | 6.22 | 5.13 | 0.009 | 13 |
| *B. Controls > guitarists for beatbox > guitar* | | | | | | | |
| L inferior frontal gyrus (pars opercularis) | **-50** | **10** | **24** | 8.13 | 6.15 | <.001 | 302 |
|  | **-46** | **8** | **32** | 6.38 | 5.23 | 0.006 |  |
|  | **-50** | **10** | **14** | 6.20 | 5.12 | 0.009 |  |
| L inferior temporal gyrus | **-50** | **-54** | **-12** | 6.90 | 5.52 | 0.001 | 49 |
| R inferior frontal gyrus (pars triangularis) | **48** | **14** | **22** | 6.62 | 5.37 | 0.003 | 71 |
| R inferior frontal gyrus (pars opercularis) | **56** | **14** | **24** | 6.11 | 5.07 | 0.012 |  |
| L postcentral gyrus | **-52** | **-36** | **58** | 6.58 | 5.34 | 0.003 | 141 |
|  | **-46** | **-38** | **48** | 6.36 | 5.21 | 0.006 |  |
| L supplementary motor area | **-6** | **18** | **48** | 6.34 | 5.20 | 0.006 | 14 |
| R cerebellum (VI) | **22** | **-68** | **-24** | 5.84 | 4.90 | 0.024 | 2 |
| L midcingulate cortex | **0** | **2** | **32** | 5.84 | 4.90 | 0.024 | 4 |
| R inferior temporal gyrus | **52** | **-48** | **-12** | 5.70 | 4.81 | 0.035 | 3 |

**Supplementary Table 4C.** Peak co-ordinates in gray matter regions in a two sample T-tests comparing beatbox > guitar music activity in beatboxers and guitarists, masked by regions that show a significant group x condition interaction.

| Area | X | Y | Z | T_38_ | Z | P (FWE-corrected) | Extent |
| --- | --- | --- | --- | --- | --- | --- | --- |
| *A. Guitarists > beatboxers for beatbox > guitar* | | | | | | | |
| R middle occipital gyrus | **36** | **-82** | **32** | 6.63 | 5.37 | 0.003 | 29 |
|  | **42** | **-76** | **30** | 6.01 | 5.01 | 0.014 |  |
| L middle occipital gyrus | **-38** | **-78** | **16** | 6.08 | 5.05 | 0.011 | 11 |
| R inferior parietal lobule | **60** | **-52** | **44** | 5.81 | 4.88 | 0.024 | 12 |
| *B. Beatboxers > guitarists for beatbox > guitar* | | | | | | | |
| L inferior frontal gyrus (pars opercularis) | **-50** | **10** | **24** | 10.07 | 6.99 | <.001 | 816 |
|  | **-46** | **8** | **32** | 8.17 | 6.17 | <.001 |  |
|  | **-50** | **8** | **14** | 8.06 | 6.12 | <.001 |  |
| R midcingulate cortex | **2** | **-2** | **32** | 9.47 | 6.75 | <.001 | 117 |
| L Postcentral gyrus | **-64** | **-18** | **22** | 9.13 | 6.60 | <.001 | 202 |
| L Postcentral gyrus | **-52** | **-38** | **18** | 8.56 | 6.35 | <.001 | 33 |
| R superior temporal gyrus | **66** | **-28** | **16** | 8.27 | 6.22 | <.001 | 130 |
|  | **60** | **-32** | **10** | 7.13 | 5.65 | 0.001 |  |
| R inferior frontal gyrus (pars triangularis) | **44** | **16** | **24** | 8.00 | 6.09 | <.001 | 305 |
| R inferior frontal gyrus (pars opercularis) | **52** | **16** | **26** | 7.93 | 6.06 | <.001 |  |
| R inferior temporal gyrus | **52** | **-50** | **-14** | 7.94 | 6.06 | <.001 | 30 |
| L Postcentral gyrus/ L inferior parietal lobule | **-52** | **-34** | **58** | 7.32 | 5.74 | <.001 | 390 |
|  | **-58** | **-28** | **54** | 6.87 | 5.51 | 0.001 |  |
|  | **-62** | **-24** | **44** | 6.44 | 5.26 | 0.004 |  |
| L inferior temporal gyrus | **-50** | **-52** | **-16** | 7.26 | 5.72 | <.001 | 90 |
| L supplementary motor area | **-4** | **4** | **64** | 7.22 | 5.70 | 0.001 | 124 |
| R Cerebellum (VI) | **28** | **-60** | **-24** | 7.16 | 5.66 | 0.001 | 71 |
| L Cerebellum (VI) | **-22** | **-62** | **-26** | 7.09 | 5.62 | 0.001 | 49 |
|  | **-66** | **-20** | **6** | 6.53 | 5.32 | 0.003 | 14 |
| R Precentral gyrus | **58** | **4** | **42** | 6.32 | 5.19 | 0.006 | 30 |
| L inferior frontal gyrus (pars triangularis) | **-46** | **36** | **16** | 6.01 | 5.01 | 0.014 | 26 |

**Supplementary Table 5.** Independent Component Analysis

Coordinates for each local maxima within significant clusters of activity (Z > 3.1, cluster extent > 10 voxels) are given for the components shown in Figure 2. PCC, Posterior cingulate cortex; SMG, supramarginal gyrus. For each component, the percentage of the total variance in the data explained by that component is given in parentheses.

| z | MNI coordinates  (x, y, z) | | | Label |
| --- | --- | --- | --- | --- |
| *C1: Auditory (3.8%)* | | | | |
| 32.3 | 48 | -16 | 2 | R Heschl’s gyrus extending down superior temporal gyrus |
| 34.5 | -58 | -16 | 2 | L planum temporale extending down superior temporal gyrus |
| 7.46 | -26 | -62 | -26 | L cerebellum lobule VI |
| 10.9 | 4 | -38 | -10 | Brain stem |
| 7.62 | 28 | -62 | -26 | R cerebellum lobule VI |
| 6.47 | -24 | -66 | -56 | L cerebellum lobule VIIIa |
| 5.98 | 54 | -2 | 44 | R precentral gyrus |
| 4.89 | 22 | -68 | -50 | R cerebellum lobule VIIIa |
| 5.81 | -54 | -10 | 46 | L precentral gyrus |
| 5.42 | -6 | -36 | -46 | Brain Stem |
| *C2: Medial Visual (3.4%)* | | | | |
| 26.9 | 0 | -80 | 16 | Precuneus, supracalcarine cortex |
| 6.14 | -46 | -12 | 50 | L precentral cortex |
| 6.59 | 36 | -16 | 40 | R precentral cortex |
| 4.64 | 4 | 12 | 36 | Anterior cingulate gyrus |
| 4.26 | -38 | 8 | 2 | L insular cortex |
| 4.28 | 38 | 10 | 6 | R insular cortex |
| 4.54 | 38 | -14 | 16 | R insular cortex |
| 4.44 | 2 | -24 | 26 | PCC |
| 4.15 | -10 | -18 | 40 | PCC |
| 4.06 | -36 | -14 | 14 | L insular cortex |
| *C3: Right fronto-temporo-parietal (3.3%)* | | | | |
| 14.8 | 34 | 16 | 54 | R middle frontal gyrus |
| 20.9 | 44 | -64 | 48 | R superior lateral occipital cortex |
| 11.9 | 62 | -42 | -12 | R middle temporal gyrus, temporo-occipital part extending into inferior temporal gyrus |
| 11.5 | -38 | -68 | -44 | L Cerebellum Crus II |
| 10.9 | 2 | -36 | 34 | R PCC |
| 8.22 | -42 | -68 | 46 | L superior lateral occipital cortex |
| 5.89 | -44 | 52 | -8 | L frontal pole |
| 3.86 | 2 | -22 | 68 | R precentral gyrus, medial |
| 4.66 | 34 | 20 | -6 | R anterior insular cortex |
| *C4: Left fronto-temporo-parietal (3.2%)* | | | | |
| 14.2 | -50 | 28 | 22 | L inferior & middle frontal gyrus |
| 14.3 | -48 | -52 | 52 | L angular gyrus & posterior SMG |
| 13.4 | -60 | -46 | -12 | L middle temporal gyrus, temporo-occipital part extending into inferior temporal gyrus |
| 10.4 | 26 | -68 | -30 | R Cerebellum Crus I |
| 4.33 | 38 | -58 | 40 | R superior lateral occipital cortex |
| 3.79 | -14 | 6 | 10 | L caudate nucleus |
| 4.06 | 48 | 36 | 18 | R frontal pole |
| 3.94 | 56 | -46 | -12 | R inferior temporal cortex |
| 4.11 | -8 | -16 | 8 | L Thalamus |
| *C5: Lateral visual (3.2%)* | | | | |
| 15.2 | 20 | -64 | 58 | R superior lateral occipital cortex |
| 6.47 | 26 | -8 | 52 | R precentral gyrus |
| 5.84 | -24 | -4 | 56 | L superior frontal gyrus, precentral gyrus |
| 5.34 | 48 | 6 | 26 | R precentral gyrus, inferior frontal gyrus |
| 3.82 | 28 | -78 | -38 | R cerebellum Crus II |
| *C6: Sensorimotor (3.2%)* | | | | |
| 16.7 | 0 | -12 | 50 | Supplementary motor cortex extending to pre- and post-central gyri bilaterally |
| 3.72 | -14 | -62 | -22 | L Cerebellum Lobule VI |
| 3.57 | 18 | -66 | -24 | R Cerebellum Lobule VI |
| 3.86 | 28 | -16 | -22 | R anterior parahippocampal gyrus |
| *C7: Default mode network (3.2%)* | | | | |
| 12.5 | 0 | 58 | 12 | Medial prefrontal cortex, superior frontal gyrus & paracingulate gyrus |
| 7.05 | -62 | -12 | -20 | L posterior middle temporal gyrus |
| 6.47 | 62 | -8 | -20 | L anterior middle temporal gyrus |
| 9.07 | 0 | -50 | 28 | PCC and precuneus |
| 6.79 | -48 | -64 | 24 | L superior lateral occipital cortex |
| 7.02 | -30 | -82 | -36 | L cerebellum Crus I & II |
| 6.34 | 54 | -64 | 24 | R superior lateral occipital cortex |
| 6.23 | 28 | -82 | -34 | R cerebellum Crus I & II |
| 5.07 | 22 | -20 | -16 | R hippocampus |
| 4.25 | 36 | -60 | -28 | R cerebellum Crus I |
| 4.76 | -22 | -18 | -16 | L hippocampus |
| 3.89 | -30 | -48 | -26 | R fusiform cortex, posterior division |
| 3.66 | 0 | -18 | 38 | PCC |
| *C8: Default mode network (3.0%)* | | | | |
| 14.7 | 2 | -66 | 30 | Precuneous |
| 13.1 | 42 | -72 | 34 | R superior lateral occipital cortex |
| 11.8 | -36 | -82 | 32 | L superior lateral occipital cortex |
| 6.61 | 2 | 54 | -10 | Medial prefrontal cortex |
| 7.31 | 26 | 30 | 44 | R middle frontal gyrus, extending to frontal pole |
| 6.44 | -24 | 26 | 36 | L middle frontal gyrus, extending to frontal pole |
| 5.68 | 54 | -6 | -18 | R anterior middle temporal gyrus |
| 4.27 | -8 | -16 | 10 | L thalamus |
| 4.09 | 6 | -16 | 8 | R thalamus |
| 3.95 | -22 | 62 | 6 | L Frontal Pole |
| 4.05 | -8 | -52 | -48 | R cerebellum lobule IX |
| 3.68 | -52 | -2 | -22 | L anterior middle temporal gyrus |
| *C9: Executive control (2.9%)* | | | | |
| 13.5 | 32 | 50 | 22 | R frontal pole |
| 9.86 | -38 | 12 | -2 | L insular cortex |
| 9.34 | -36 | -54 | -34 | L cerebellum crus I |
| 8.02 | 38 | -56 | -32 | R cerebellum crus I |
| 6.76 | 58 | -34 | 36 | R anterior SMG |
| 6.19 | 20 | 8 | 64 | R superior frontal gyrus, extending to cingulate cortex and precuneus |
| 6.14 | -62 | -36 | 30 | L anterior SMG |
| 4.86 | -20 | 8 | 62 | L superior frontal gyrus, extending to cingulate cortex and precuneus |
| 4.53 | 2 | -76 | -18 | R cerebellum vermis VI |
| 3.54 | -26 | -4 | 2 | L putamen |
| *C10: Lateral visual + occipital pole (2.9%)* | | | | |
| 19.7 | 26 | -98 | 0 | R occipital pole, extending to lateral occipital cortex and L occipital pole |
| 5.14 | 6 | -14 | 8 | R thalamus |
| 3.9 | 20 | 8 | 20 | R caudate |
| *C11: Higher-level sensorimotor (2.7%)* | | | | |
| 10.6 | 46 | 12 | 26 | R inferior frontal gyrus, extending to precentral gyrus |
| 7.84 | 58 | -46 | 14 | R angular gyrus extending to SMG & parietal operculum |
| 7.14 | -48 | 10 | 24 | L inferior frontal gyrus, extending to precentral gyrus |
| 6.07 | -62 | -20 | 22 | L postcentral gyrus, extending to SMG, parietal operculum and angular gyrus |
| 6.31 | 2 | 10 | 42 | Supplementary motor cortex + ACC |
| 4.34 | -24 | 2 | -16 | L amydala |
| 4.14 | 8 | -14 | 6 | R thalamus |
| *C12: Temporal opercular (2.6%)* | | | | |
| 8.74 | -58 | -54 | 30 | L angular gyrus, extends to superior temporal sulcus |
| 6.41 | -6 | 20 | 66 | R superior frontal gyrus, medial surface |
| 7.67 | 58 | -48 | 32 | R angular gyrus, extends to superior temporal sulcus |
| 6.79 | -32 | 20 | -18 | L orbitofrontal cortex |
| 5.33 | 54 | 32 | -12 | R orbitofrontal cortex |
| 4.32 | -54 | 8 | -32 | L temporal pole |
| 4.69 | -44 | 12 | 52 | L middle frontal gyrus |
| 4.9 | 52 | 14 | -40 | R temporal pole |
| 5.2 | 24 | 54 | 24 | R frontal pole |
| 4.05 | -8 | -50 | 32 | PCC |
| 4.17 | 0 | -20 | 38 | PCC |
| 3.54 | -8 | 64 | -20 | L frontal pole |
| 3.65 | 44 | 10 | 50 | R middle frontal gyrus |
| 3.98 | -20 | -74 | -34 | L cerebellum crus I |
| 3.74 | 42 | 24 | 42 | R middle frontal gyrus |
| *C13: Movement related noise in ventricles +the edges of the cerebellum (2.1%)* | | | | |

**Supplementary Table 6.**

Means and standard deviations of beatbox > guitar activity in beatboxers, guitarists, and non-musicians, for the six networks that show significant group x condition interaction

| *Beatbox > Guitar* | *Nonmusicians* | *Guitarists* | *Beatboxers* | *Group x condition* |
| --- | --- | --- | --- | --- |
| C1 | 1.5  (.66) | .32  (.68) | 2.25  (.64) | *F*(2,57)=43.4,*p*<.001 |
| C4 | -.32  (1.04) | -1.71  (1.17) | -.23  (.92) | *F*(2,57)=12.5,*p*<.001 |
| C6 | -.11  (1.0) | -1.09  (.87) | .47  (1.07) | *F*(2,57)=12.8,*p*<.001 |
| C8 | -.31  (1.02) | 1.01  (1.24) | -1.08  (1.43) | *F*(2,57)=14.5,*p*<.001 |
| C9 | -.73  (1.25) | .14  (1.28) | -1.4  (1.32) | *F*(2,57)=7.2,*p*=.002 |
| C11 | -.85  (.84) | -1.67  (1.87) | -3.13  (1.91) | *F*(2,57)=43.9,*p*<.001 |

**Supplementary Table 7**

We report the results of one-way ANOVAs on each of these variables, with follow-up group comparisons reported when the overall group difference is significant (p <.05).

|  | *Nonmusicians* | *Guitarists* | *Beatboxers* | *Group difference* | *NM vs. G* | *NM vs. B* | *G vs B* |
| --- | --- | --- | --- | --- | --- | --- | --- |
| Age | 27.8 (8.9) | 30.0 (7.8) | 26.8 (5.8) | *F*(2,57) = .93,  *p* =.40 | Group difference n.s. | | |
| *Age of onset* | n/a | 11.2 (2.3) | 14.0 (3.8) | n/a | | | *t*(38)=2.82, *p*=.008 |
| *Musical training (years)* | 0.03 (0.1) | 5.9 (3.9) | 3.1 (4.1) | n/a | | | *t*(38)=2.15, *p*=.038 |
| Professional Experience (years) | n/a | 8.7 (7.3) | 8.2 (5.3) | n/a | | | *t*(38)=.21, *p*=.84 |
| Amateur Experience (years) | n/a | 11.6 (7.2) | 11.3 (8.0) | n/a | | | *t*(22)=.13, *p*=.90 |
| *Cognitive Tests* | | | |  |  |  |  |
| Pure Tone Average | 1.3 (4.0) | 1.7 (3.6) | 2.7 (5.1) | *F*(2,57) = .58,  *p* =.56 | Group difference n.s. | | |
| Matrix Reasoning Ability (Scaled) | 60.4 (4.7) | 57.9 (6.9) | 58.2 (7.3) | *F*(2,57) = .86,  *p* =.42 | Group difference n.s. | | |
| Working memory | 12.8 (3.2) | 12 (3.2) | 11.7 (3.5) | *F*(2,57) = .54,  *p* =.59 | Group difference n.s. | | |
| *Perception Tests* | | | |  |  |  |  |
| *Metric judgment* | 25.2 (5.5) | 29.9 (0.4) | 27.2 (3.8) | *F*(2,56) = 7.45, *p*<.001 | *t*(19.1)=3.8, *p*<.001 | *t*(37)=1.36, *p*=.183 | *t*(18.3)=3.04, *p*=.007 |
| *Rhythm discrimination* | 24.1 (3.2) | 27.1 (2.5) | 26.2 (2.5) | *F*(2,56) = 6.27, *p*=.004 | *t*(38)=3.33, *p*=.002 | *t*(37)=2.24, *p*=.031 | *t*(37)=1.18, *p*=.244 |
| *Frequency discrimination threshold* | 10.6 (7.3) | 4.8 (4.4) | 8.0 (3.9) | *F*(2,55) = 5.6, *p*=.006 | *t*(37)=3.00, *p*=.005 | *t*(36)=1.35, *p*=.187 | *t*(37)=2.40, *p*=.021 |
| Duration discrimination threshold | 28.6 (10.8) | 25.3 (13.2) | 27.0 (9.3) | *F*(2,56) = .446, *p*=.643 | Group difference n.s. | | |
| *Musicality: Goldsmiths Musical Sophistication Index* | | | |  |  |  |  |
| *Active Engagement* | 32.4 (11.6) | 50.3 (4.2) | 50.5 (6.4) | *F*(2,57) = 33.5, *p<.001* | *t*(23.9)=6.49, *p*<.001 | *t*(29.6)=6.1, *p*<.001 | *t*(38)=.087, *p*=.931 |
| *Perceptual abilities* | 42.6 (6.6) | 56.8 (4.0) | 51.8 (7.8) | *F*(2,57) = 25.85, *p<.001* | *t*(38)=8.23, *p*<.001 | *t*(38)=4.01, *p*<.001 | *t*(28.3)=2.57, *p*=.016 |
| *Musical Training* | 13.3 (6.3) | 43.1 (5.1) | 34.9 (6.3) | *F*(2,57) = 135.07, *p<.001* | *t*(38)=16.4, *p*<.001 | *t*(38)=10.88, *p*<.001 | *t*(38)=4.52, *p*<.001 |
| *Emotions* | 31.8 (4.1) | 37.2 (3.5) | 35.8 (4.6) | *F*(2,57) = 9.35, *p<.001* | *t*(38)=4.46, *p*<.001 | *t*(38)=2.89, *p*=.006 | *t*(38)=1.09, *p*=.284 |
| *Singing Abilities* | 22.0 (8.0) | 38.1 (6.1) | 35.1 (8.8) | *F*(2,57) = 24.94, *p<.001* | *t*(38)=7.2, *p*<.001 | *t*(38)=4.96, *p*<.001 | *t*(38)=1.26, *p*=.216 |
| *General Sophistication* | 55.1 (14.1) | 105.9 (9.0) | 100.6 (13.6) | *F*(2,57) = 100.41, *p<.001* | *t*(38)=13.57, *p*<.001 | *t*(38)=10.37, *p*<.001 | *t*(38)=1.44, *p*=.158 |

**Supplemental Data: Appendix 1**

**ROI identification for each participant**

We used a threshold of p<.05 FWE to identify clusters within the Mouth > Rest contrast in each participant. Clusters that were spatially localised to the sensorimotor cortex in this contrast were identified visually and labelled left and right mouth ROIs respectively. Using the SPM toolbox marsbar, we saved identified clusters and extracted mean beta values for the [beatbox > guitar music] contrast in these clusters for each participant. One participant did not have any mouth > rest activity at this threshold (however, this participant did have significant activity for the mouth > rest at p<.001 uncorrected). To maintain the same thresholds across participants, this participant was excluded from the ROI analysis. For an additional 2 participants, mouth > rest clusters could not be identified in the right hemisphere at this threshold. Consequently, no beatbox > guitar beta values for the right hemisphere cluster were extracted in these participants.

Clusters lying within the sensorimotor cortex for the Hand > Rest contrast were also identified using a threshold of p<.05 FWE. These were visually inspected and labelled left and right hand ROIs respectively. Again, using the SPM toolbox marsbar, we saved identified clusters and extracted mean beta values for the [beatbox > guitar music] contrast in these clusters for each participant. Four of our sixty participants, including the one highlighted above, did not show significant hand > rest activity at p<.05 FWE and were consequently excluded from these individual analyses. Three participants had large, spatially unspecific clusters of hand > rest activity that extended over both hemispheres, these participants were excluded from the analyses as refining the clusters would have required changing the threshold, and therefore biasing our results. Six participants did not have identifiable hand>rest clusters in the right hemisphere, and four participants did not have identifiable hand>rest clusters in the left hemisphere. We therefore only extracted beta values for beatbox > guitar music from one hemisphere for these participants.

Using SPSS, we conducted a 2 x 2 x 3 ANOVA on the mean beta values to determine if hemisphere (left/right), region (mouth/hand), and group (beatboxers/guitarists/non-musicians) modulated beatbox > guitar activity within these ROIs.

**Results from individual ROIs**

We assessed whether hemisphere (left/right), effector (hand/mouth region) and group (non-musician, beatboxer, guitarist) modulated [beatbox > guitar activity] in the 4 ROIs we extracted as described above. Given the exclusions described above, we retained data from 16 guitarists, 13 beatboxers and 12 non-musicians for this analysis. We observed a significant influence of group, *F*(2,38)=10.187, *p*<.001, hemisphere, *F*(1,38)=10.444, *p*=.003, and a trend for an interaction between effector region x group, *F*(2,38)=2.753, *p*=.076. The main effect of group reflected a cross over effect of expertise. Guitarists differed from both beatboxers (*p*<.001) and non-musicians (*p*=.049), showing a guitar > beatbox music preference. Beatboxers also differed from non-musicians (*p*=.029) as they had greater beatbox > guitar activity. The effector region x group interaction was driven by the guitarists showing a trend for increasing their guitar > beatbox preference in the hand region, *t*(15)=2.036, *p*=.06, whereas both the beatboxers, *t*(12)=.973, *p*=.35, and the non-musicians, *t*(11)=.328, *p*=.75, did not show a modulation of beatbox > guitar activity by region.

Given the lack of a hemisphere by group interaction, we averaged beatbox > guitar activity across the left and right mouth and hand ROIs respectively to derive a regionally-specific Mouth beatbox > guitar activity score and a Hand beatbox > guitar activity score. For participants where only one of the left/right clusters could be identified, we used the beatbox > guitar activity from the hemisphere that could be identified. This allowed us to include more participants in this analysis. Again, in the hand regions, beatbox > guitar activity was significantly modulated by group membership. Guitarists showed a guitar > beatbox preference (see Supplementary Figure 2), which was significantly different to the preference showed by beatboxers (*p*<.001), and non-musicians (*p*=.001). In the mouth region, beatboxers showed a beatbox > guitar music preference (see Supplementary Figure 2), which was significantly different from the guitarists (*p*<.001) and from non-musicians (*p*=.047).

**Supplementary Figure 2.** depicts the results of univariate ROI analyses, where mean beta values for left and right hand and mouth regions were extracted for beatbox > guitar music in each participant. Positive values indicate more activity for beatboxing, whereas negative values suggest more activity for guitar music. Error bars depict +/- 1 standard error of the mean. Each data point represents an individual participant. Non-musicians (NM) are represented by filled circles, beatboxers (BB) by filled squares and guitarists (G) using filled triangles. These graphs clearly show that in both hand and mouth regions, guitarists show greater activity for guitar music and beatboxers show greater activity for beatboxing. The specific ROIs we sampled from were derived from individual activity for hand and mouth movements.

**Discussion**

While we confirm the expertise-specific responses we observed when using the spherical ROIs, individualized ROIs also revealed that guitarists show a guitar > beatbox music preference in mouth areas, and that beatboxers show a beatbox > guitar music preference in hand areas. The former result might be because the mouth > silence representations tended to be quite large across individuals, and by not constraining the ROIs in any way, these ROIs may include voxels that are sensitive to hand activity (Supplementary Figure 3).

On the other hand, hand representations tended to be more spatially constrained. One explanation for cross-over expertise effects in hand regions may be because beatboxers do typically use their hands in time with music as they perform. However, an alternative explanation is that this region is flexibly used to simulate rhythm (Grahn and McAuley 2009), and therefore both groups of musicians would be likely to recruit this region to make predictions about upcoming beats for their specific musical style.

Yet, it is important to be cautious when interpreting these results. The individualized ROI analysis approach is limited by the fact that ROIs are highly variable across the set threshold, with some participants showing strong activity across a large set of voxels at this threshold, and others showing almost no activity. As highlighted above, some ROIs derived using this method tend to lie outside of sensorimotor cortex, taking in voxels over superior temporal cortex (Supplementary figure 3). In addition, when using this individualized approach, we cannot include data from all participants, therefore reducing our sensitivity to any group differences. Visual inspection of summed ROIs by group also suggested that the size of the ROIs did not vary much across the three groups (Supplementary figure 3), suggesting that biases in the size of hand or mouth representation were unlikely to be the cause of any group differences. Consequently, we chose to avoid these constraints in the main analysis by using a spherical ROI across participants.

**Supplementary Figure 3.** depicts the distribution of hand > rest and mouth > rest ROIs across the three groups. A heat scale is used to depict the number of participants with activity in a region, regions in yellow have 6+ participants.
